# Long-term water quality assessment and trophic status trends in Dobromierz, Lubachów and Sosnówka drinking water reservoirs in southwestern Poland

**DOI:** 10.1038/s41598-025-94219-3

**Published:** 2025-03-21

**Authors:** Magdalena Szewczyk, Paweł Tomczyk, Mirosław Wiatkowski

**Affiliations:** 1 Provincial Fund for Environmental Protection and Water Management in Opole, Opole, Poland; 2 Institute of Environmental Engineering, Wrocław University of Environmental and Life Sciences, Wrocław, Poland

**Keywords:** Water security, Dam reservoirs, Drinking water reservoirs, Eutrophication, Water management, Water quality indices, Ecological indicators, Environmental monitoring, Environmental impact

## Abstract

The aim of the article is to assess the water quality in three drinking water reservoirs in southwestern Poland, i.e. in Dobromierz, Lubachów and Sosnówka, taking into account two classification methods, to determine the trophic status and water quality indicators of 3 research objects, to analyze potential sources of anthropogenic impact on catchments in order to identify the causes of deterioration of the trophic status of reservoirs. When analyzing the results of water quality indicators for the period 1992–2022, reference was made to two classification methods: the new one, in force from 1st January 2022, and the old one, valid from 22nd October 2014 to 31st December 2021. Due to changes in the monitoring system resulting from the implementation of the assumptions of the Water Framework Directive, the scope of water quality analyses has been reduced from 23 to 9 parameters. The need to use two methods of classification of surface water bodies in the article is aimed at an in-depth analysis of water quality using an extended set of indicators. Due to limitations in data access for some indicators in individual years, the analyses conducted are of indicative nature. The overall water quality expressed by the average value of the calculated 6 water quality indices for the period 1992–2022 was moderate for the Dobromierz and Lubachów reservoirs and good for the Sosnówka reservoir. According to the new classification, the average water quality was in classes I or II. The article addresses the issue of eutrophication of water intended for consumption by determining the trophic status of the objects based on 4 trophic indices. In the years 1992–2022, the overall trophic status of the reservoirs oscillated between mesotrophic and eutrophic. The statistical analysis showed a high variability of physicochemical parameters of water. The parameters that worsened the ecological status of water in the reservoirs were NO_3_^–^N, BOD_5_, NH_4_-N, TOC and TN. Taking into account information on existing sources of anthropogenic impact on the catchment area allowed for the explanation of potentially possible causes of deterioration of the trophic status of waters. The studied reservoirs are facilities at risk of eutrophication and the main pressure is caused by surface runoff of nutrients and unorganized water and wastewater management in the catchment area. Long-term monitoring of water quality indicators in drinking water reservoirs is needed in order to develop adaptive measures to environmental changes in the catchment area to ensure the safety and reliability of the entire water supply system.

## Introduction

The importance of water for humans and the natural environment is emphasized in many political, social and economic debates. This is primarily due to the difficulties in providing good and clean water to all users, which exist now or may arise in the future^1,2^. The development of industry and the wide use of agrochemicals have subjected artificial water reservoirs to increased stress, thus causing a deterioration of their water quality^3–5^. According to the United Nations, 56% of domestic wastewater flows were safely treated in 2020 worldwide (data from 128 countries representing 80% of the world’s population). However, progress remains uneven around the world, due to large disparities in regional proportions of safely treated domestic wastewater, which threatens water quality in lakes^6^.

Changes in water quality in reservoirs may adversely affect the functioning of facilities, especially in the case of reservoirs storing drinking water^7–16^. The sources of internal water pollution in reservoirs are accumulated bottom sediments releasing, among others, phosphorus in the pelagic zone^10,14,16^ and the phenomenon of seasonal stratification causing the mobility of elements and deoxygenated bottom waters^11,13^. The sources of external water pollution in reservoirs are the supplied suspension (organic material such as algae and inorganic material such as sediment particles) and nutrients from the catchment area, which accelerate the rate of the eutrophication process^17^. Eutrophication caused by the inflow of large amounts of organic substances, especially compounds rich in phosphorus and nitrogen, affects the increase in the fertility of the reservoir and the development of undesirable cyanobacteria, diatoms and green algae. Their excessive growth, called water bloom, causes a significant deterioration in water quality (especially oxygen and light conditions), and the reservoir itself cannot perform its intended functions^1,18–24^. Eutrophicated water may also exhibit toxic properties due to the release of metabolic products from cyanobacteria^21^.

In many regions of the world, the problem of point source pollution of water reservoirs, mainly related to insufficient wastewater treatment, is effectively solved by building new and modernizing existing wastewater treatment plants, as well as expanding wastewater systems. Pollution from diffuse sources is the main and remaining cause of water quality problems in reservoirs on a local, regional and global scale^14,15,25^.

A global study of over 2,500 water bodies showed that 63.1% of them were eutrophic and were concentrated mainly in East Asia, Central Africa and Central and Southeastern North America^22,23,26^. This is confirmed by water quality studies already conducted on specific sites, including the Ontario Reservoir^27^, the Nasser Reservoir in Egypt^28^, the Taihu Reservoir in China^29^, the Keban Reservoir in Turkey^30^, the Ypacaraí Reservoir in Paraguay^31^, the La Vega Reservoir in Mexico^32^, on Lake Cedrino in Italy^33^, on the Rappbode and Haseł reservoirs in Germany^34^, on Lake Albufera in Spain^35^, on the Marateca reservoir in Portugal^36^ or on the Švihov reservoir in the Czech Republic^37^.

Poor water quality in reservoirs serving the population causes technological problems and increases the costs of water treatment. Toxins that appear in connection with harmful blooms of cyanobacteria and increased amounts of suspended solids require the use of complex and expensive technologies for drinking water treatment^22,38^.

Studies confirm the high sensitivity of the ecological status of reservoirs to land use in their catchment areas. In built–up or agricultural catchment areas, an increase in the concentrations of undesirable elements (including Ca, Sr, Mn, Al, Ni, Zn) is observed to a large extent^7,8,16–18^. In the case of water reservoirs located in ecologically sustainable and forested areas, such risk is lower^9^.

Over the past few decades, a network of lake water quality monitoring has been established at local agencies or national water boards, especially in developed countries, but information on inland lakes is still scattered and heterogeneous, making it difficult to detect changes in water quality on a global scale^6^. In the United States, water quality is regulated by the Safe Drinking Water Act (SDWA)^39^. The SDWA authorized the U.S. Environmental Protection Agency (EPA) to develop minimum parameters for drinking water and to establish appropriate standardized analytical methods. This key piece of legislation underpins the EU’s commitment to improving the status of Europe’s waters. The aim of EU water policy is to ensure that all users have access to good quality water in sufficient quantity and to achieve good chemical and quantitative status of EU waters by the end of each planning cycle, currently by 2027^40,41^.

The main EU law on drinking water is the Drinking Water Directive. It concerns access to water intended for human consumption and its quality in order to protect human health. Member States are obliged to report data on the quality of water intended for human consumption and update it annually^42^.

Reporting of data on water quality in reservoirs is carried out as part of the monitoring of surface water quality (SWQ) conducted by member states. Monitoring is carried out based on the JDW classification in force since 1 January 2022. This classification is based, among others, on 9 physicochemical parameters of water. In the period from 22 October 2014 to 31 December 2021, the SWQ classification based on 23 indicators was in force. Due to changes in the monitoring system resulting from the implementation of the assumptions of the Water Framework Directive, the scope of analyses conducted has been limited only to parameters for the purposes of effective management of surface water quality^43,44^.

Monitoring and assessment of the trophic status of reservoir waters is based on integrated indicators^22^. The trophic status of standing waters is determined based on indicators such as the Carlson Index (TSI)^45^ or the trophic level index (TLI)^46–48^. Also important is the classification of Vollenweider and Kereks (1982)^49^, based on the phosphorus and nitrogen loads in water bodies, which is also widely used (Istvánovics, 2009)^50^. In turn, characteristic indicators for assessing the state of water quality are: Oregon Water Quality Index (OWQI), Dinius Water Quality Index (DWQI), Overall Index of Pollution (OIP), Indian Central Control Board Water Quality Index (CPCB WQI), Universal Water Quality Index (UWQI) and The National Sanitation Foundation Water Quality Index (NSF WQI)^51–58^. Water quality indicators enable standardization of assessments of individual water quality parameters depending on the adopted nomenclature: physical, chemical, biological and hydromorphological parameters. The results obtained for the relevant indicators are helpful for water users, authorities responsible for water management and monitoring, scientists and practitioners who can identify potential sources of pollution and develop mitigation strategies^57–59^.

The objectives of the article are: 1) to assess the water quality in three drinking water reservoirs in southwestern Poland, i.e. in Dobromierz, Lubachów and Sosnówka, taking into account two classification methods (the new one, in force since January 1, 2022, based on 9 assessment indicators, and the old one, valid from October 22, 2014 to December 31, 2021, based on 23 assessment indicators), 2) to determine the trophic status and water quality indicators of 3 research objects, 3) to analyze potential sources of anthropogenic impact on catchments in order to identify the causes of the deterioration of the trophic status of the reservoirs.

The issues described in the article are important from the point of view of sustainable development goals (especially SDG 6—“clean water and sanitation”)^60^, policies related to rational water management (including in water reservoirs) and in the context of supplying the population with water with appropriate utility parameters. In the face of decreasing available drinking water resources and ongoing climate change, studies on the assessment of the ecological status of drinking water reservoirs will fill the research gap.

## Materials and methods

### Study area

**Fig. 1:**
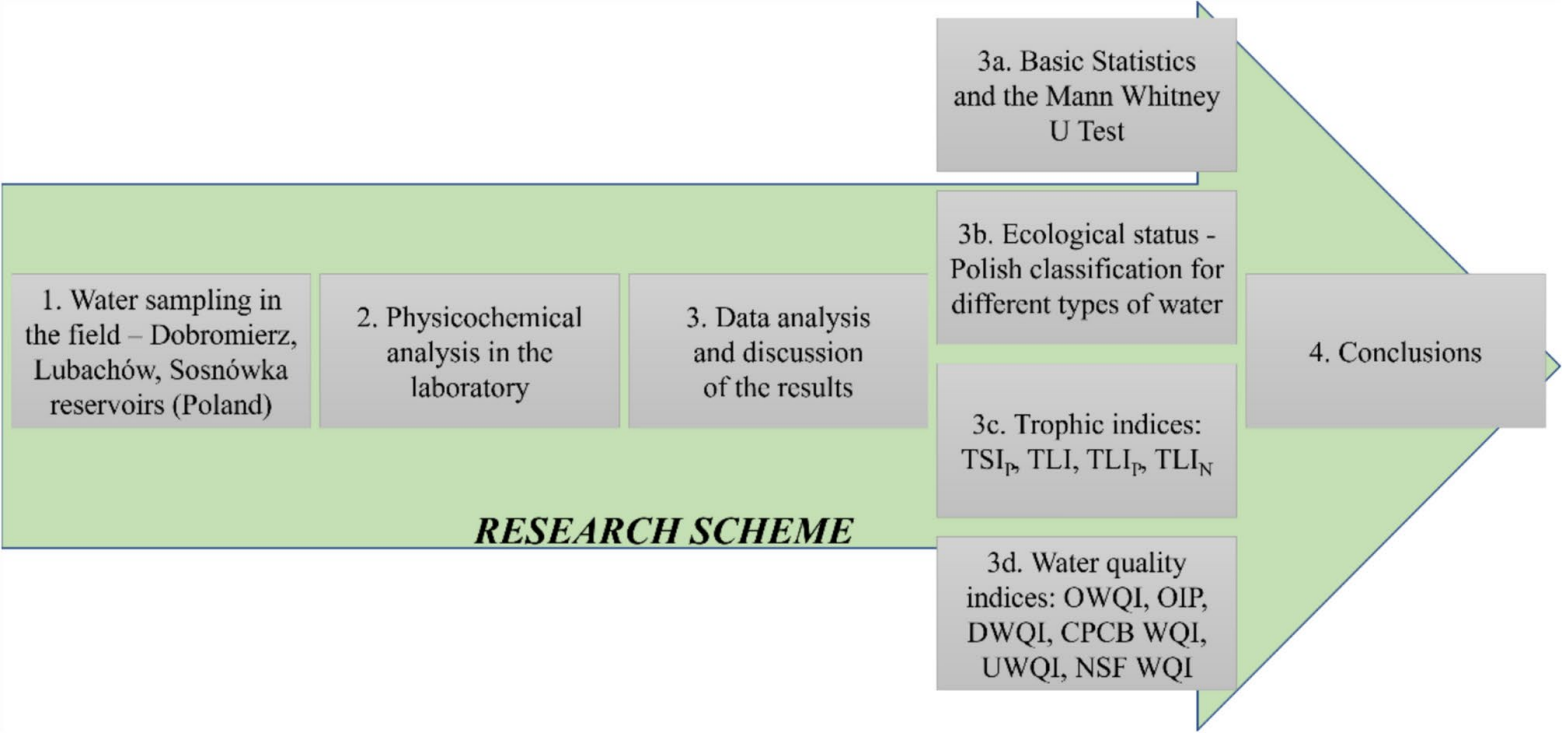
Schematic representation of the steps taken in the implementation of the article.

The article focuses on the assessment of water quality in three reservoirs: Dobromierz, Lubachów and Sosnówka. The detailed research scheme is shown in Fig. 1.

The first research object is the Dobromierz reservoir (50°54′3"N, 16°14′45"E) located in southwestern Poland (Lower Silesian Voivodeship), in the Dobromierz commune. The reservoir was built in 1986 in the 62nd km of the Strzegomka River (left–bank tributary of the Bystrzyca, a tributary of the Odra). The reservoir has a capacity of 10 million m^3^ and an area of 103 ha. The main function of the Dobromierz reservoir is to supply water for the consumption of the city of Świebodzice. Other functions of the reservoir include alimentation of flows on the Strzegomka River, flood protection of the areas located below the dam, production of electricity and fish farming^61^. The reservoir catchment area has an agricultural and forest character (52% agricultural land, 38% forest land). Its area is 8.03 km^2^. The main pressures in the reservoir catchment area are surface runoff of fertilizers from agricultural areas and municipal wastewater (diffuse sources)^62^. The share of residents using the wastewater system in the Dobromierz commune is 74.9%^63^. Wastewater from urban areas is discharged to treatment plants in Serwinów and Czernica. The problem is rural areas, which are not covered by the wastewater system. Wastewater here is mainly collected in holding reservoirs and domestic wastewater treatment plants^64^.

The Lubachów reservoir (50°45′9.10"N, 16°25′49"E) is the second research object located in southwestern Poland (Lower Silesian Voivodeship). It is located in the Walim commune, in a valley surrounded by rocky hills, mostly forested. It was built in 1917 in the 75th km of the Bystrzyca River (left tributary of the Odra). The basic parameters of the reservoir are a capacity of 9.1 million m^3^ and an area of 51 ha. The reservoir is a drinking and industrial water intake for Dzierżoniów, Pieszyce, Bielawa and adjacent towns. Additionally, it serves as a flood control and energy source. The reservoir is also partially available for recreation, hence its direct catchment area is home to recreation centers and summer cottages^65,66^. The reservoir catchment area is of an agricultural and forest character (52% forest areas, 42% agricultural areas, 5% urban areas). Its surface area is 133.09 km^2^. The main pressures in the reservoir catchment area are surface runoff of nutrients from agricultural areas and industrial and municipal wastewater (point and dispersed sources)^67^. The share of residents using the wastewater system in the Walim commune is only 40.0%^63^. Wastewater from urban areas is discharged to the treatment plant in Jugowice. The outlet of treated wastewater is located below the reservoir. In rural areas, wastewater management is disorderly, wastewater is collected mainly in holding reservoirs and domestic wastewater treatment plants^68^.

The third research object is the Sosnówka reservoir (50°49′40"N, 15°42′32"E), also located in southwestern Poland (Lower Silesian Voivodeship), in the Podgórzyn commune. It was built in 2002 in the 3rd km of the Czerwonka River and is the youngest of the analyzed reservoirs. The reservoir has a capacity of 10.9 million m^3^ and an area of 100 ha. It serves as a flood control and water supply facility for the Jelenia Góra agglomeration. The reservoir is surrounded by recreation centers and summer cottages. The reservoir catchment area is an area of forests and meadows, with small deposits of mountain peat^69^. The reservoir catchment area has an agricultural and forest character (34% agricultural land, 45% forest land, 9% urbanized areas). Its area is 6.65 km^2^. The main pressures in the reservoir catchment area are industrial and municipal wastewater (diffuse sources)^70^. The share of residents using the wastewater system in the Podgórzyn commune is 85.4%^63^. Wastewater from these areas is discharged to two container wastewater treatment plants and a treatment plant in Mysłakowice^71^.

### Water quality monitoring network

The analysed reservoirs are covered by the monitoring programme of surface water bodies (SWB) of the Lower Silesian Voivodeship, where measurements are conducted by the Voivodeship Inspectorate for Environmental Protection (WIOŚ) in Wrocław. The reservoir studies were conducted within the monitoring network:

- Operational (assessment of the state of waters at risk of failure to achieve the objectives and any changes in the state resulting from the implementation of the activities),
- Research (explanation of the reasons for failure to achieve the environmental objectives, the size and impact of pollution on the state of water, reasons for discrepancies between the results),
- Of protected areas (in the context of environmental objectives in protected areas)^72^.

Within the monitoring network, 8 measurement and control points were designated by WIOŚ, i.e. 3 points for the Dobromierz reservoir (on the river above and below the reservoir, on the reservoir), 3 points for the Lubachów reservoir (on the river above and below the reservoir, on the reservoir), 2 points for the Sosnówka reservoir (on the river below the reservoir, on the reservoir).

Table S1 in the Supplementary Material presents a list of WIOŚ measurement and control points within the Dobromierz, Lubachów and Sosnówka reservoirs^62,67,70,73^.

In the analysis monthly WIOŚ water quality data (1992–2022) were analyzes from the Dobromierz, Lubachów and Sosnówka reservoirs^73^. Measurements at the three analysed reservoirs began in 2010 (in 2012, 2018, 2019 no measurements were performed, and in the case of the Lubachów reservoir also in 2020–2022, due to changes in the monitoring system resulting from the implementation of the assumptions of the Water Framework Directive—limiting the number of measurement points within surface water bodies and the scope of analyses conducted only to parameters for the purposes of effective management of surface water quality in the light of the adopted six–year planning cycles. In the years 1992–2009, measurements were conducted only on the rivers feeding the reservoirs. Currently, the classification of water quality indicators adopted on 1 January 2022 (the so-called new classification) is in force. It is based on 9 physicochemical parameters and 34 types of surface waters^43^. In the period from 22.10.2014 to 31.12.2021, the classification was based on 23 physicochemical parameters and 41 water categories (the so-called old classification)^44^. The article presents the results of water quality according to both the new and old classification. This approach should be treated as an attempt to present an in-depth analysis of water quality using a broader set of parameters and their impact on the ecological status of the studied reservoirs. This analysis also shows how the approach to water quality management has changed over the years in the light of changes in the provisions of the WFD. Due to the lack of results in some years for the research points, as part of the monitoring conducted by WIOŚ, the analyses below should be considered indicative in nature, showing the general ecological status of waters.

**Fig. 2:**
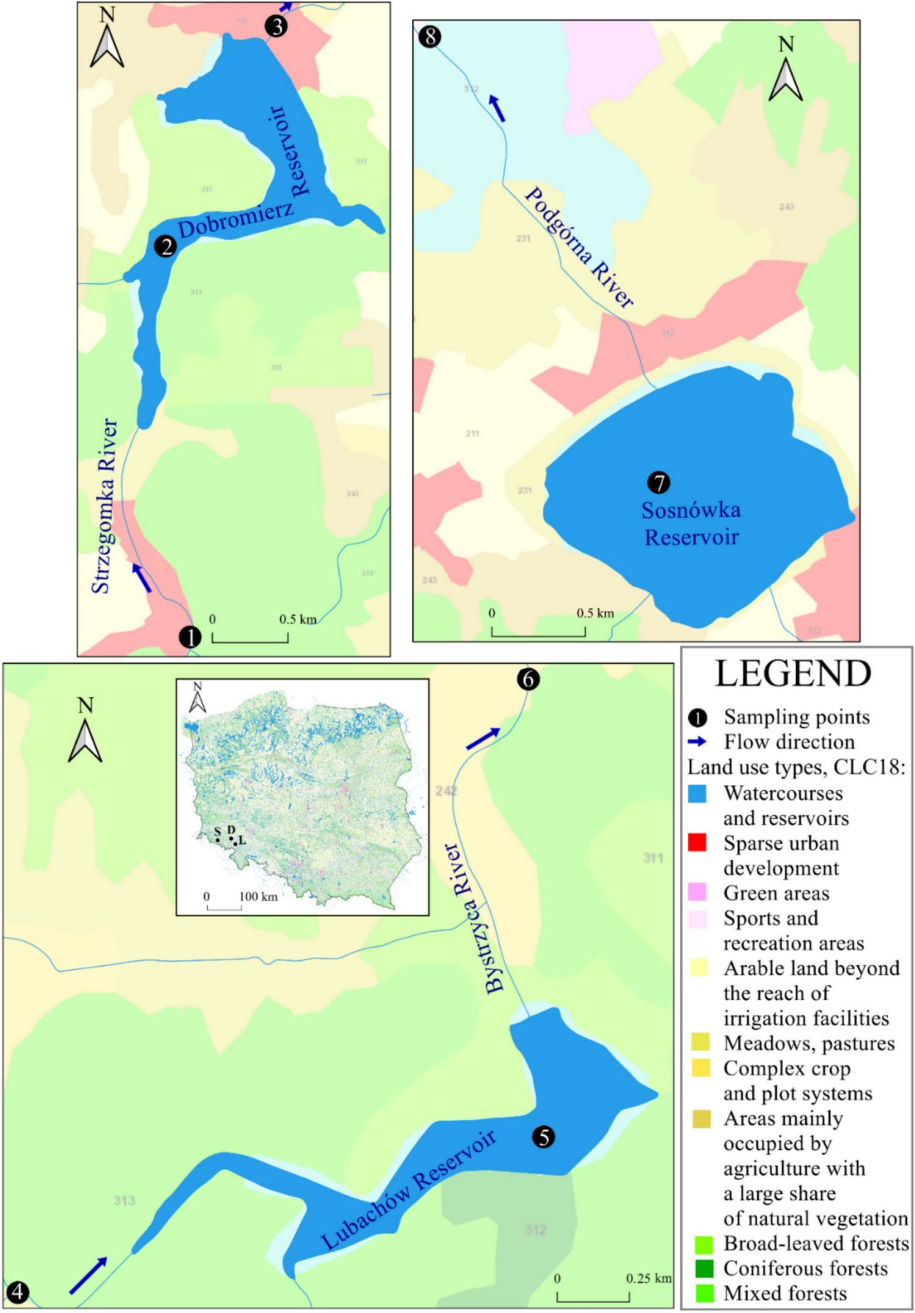
Location of the analyzed measurement and control points within the Dobromierz (1–3; D), Lubachów (4–6; L) and Sosnówka (7–8; S) water reservoirs in Poland. (map base: Corine Land Cover 2018; https://clc.gios.gov.pl/index.php/clc-2018/udostepnianie).

The location of the described measurement and control points is shown in Fig. 2.

Laboratory tests, including sampling for testing, were performed by WIOŚ research laboratories in Wrocław on behalf of the Chief Inspectorate for Environmental Protection in Warsaw (as part of the Polish State Environmental Monitoring). The tests were performed based on the methodology according to the standards included in the accreditation of the research laboratory number AB 075. Water samples were collected in accordance with the methodology according to the PN-EN ISO 5667-6:2016-12^74–76^. In the Supplementary Material presented the sampling methodology and the specification of laboratory methods for determining physicochemical parameters of water, which were included in the article (Table S2).

### Ecological status

As the first element of the analysis of the data obtained, the article analyzed the fulfillment of the requirements of the parameters concerning the limit values of selected physicochemical parameters of water quality and calculated the average ecological status for these parameters according to the new and old classification. The Regulation of the Minister of Infrastructure of June 25, 2021 on the classification of ecological status, ecological potential and chemical status and the method of classifying the status of surface water bodies and environmental quality standards for priority substances was used to make the assessment. Reference was made to two classifications: the new one, in force from January 1, 2022^43^ and the old one, valid from October 22, 2014 to December 31, 2021^44^. In the context of water reservoirs (points 2, 5 and 7), the classification was carried out for surface water bodies of type “L” (limnic reservoirs) and “0” (dam reservoirs), respectively. With regard to type “L” in the light of the current regulations (new classification), the same type as the river on which the reservoir was created should be used—for Dobromierz and Lubachów it is “RW_krz” (stream or small upland river on silicate substrate), and in the context of Sosnówka—“PGS” (Sudetic stream). The remaining research points were classified into the following types: a) new classification: points 1, 3, 5–6—“RW_krz” (stream or small upland river on silicate substrate), point 8—“PGS” (Sudetic stream); b) old classification: point 1—type “4” (silicate upland stream with coarse–grained substrate—western), 3, 4, 6—type “8” (small silicate upland river—western), 8—type “0” (dam reservoir). The assessment method according to the new and old classification for points in reservoirs is presented in Tables 1 and 2, and for points on rivers—in the supplementary material (Table S3, Supplementary Material).

**Table 1:**
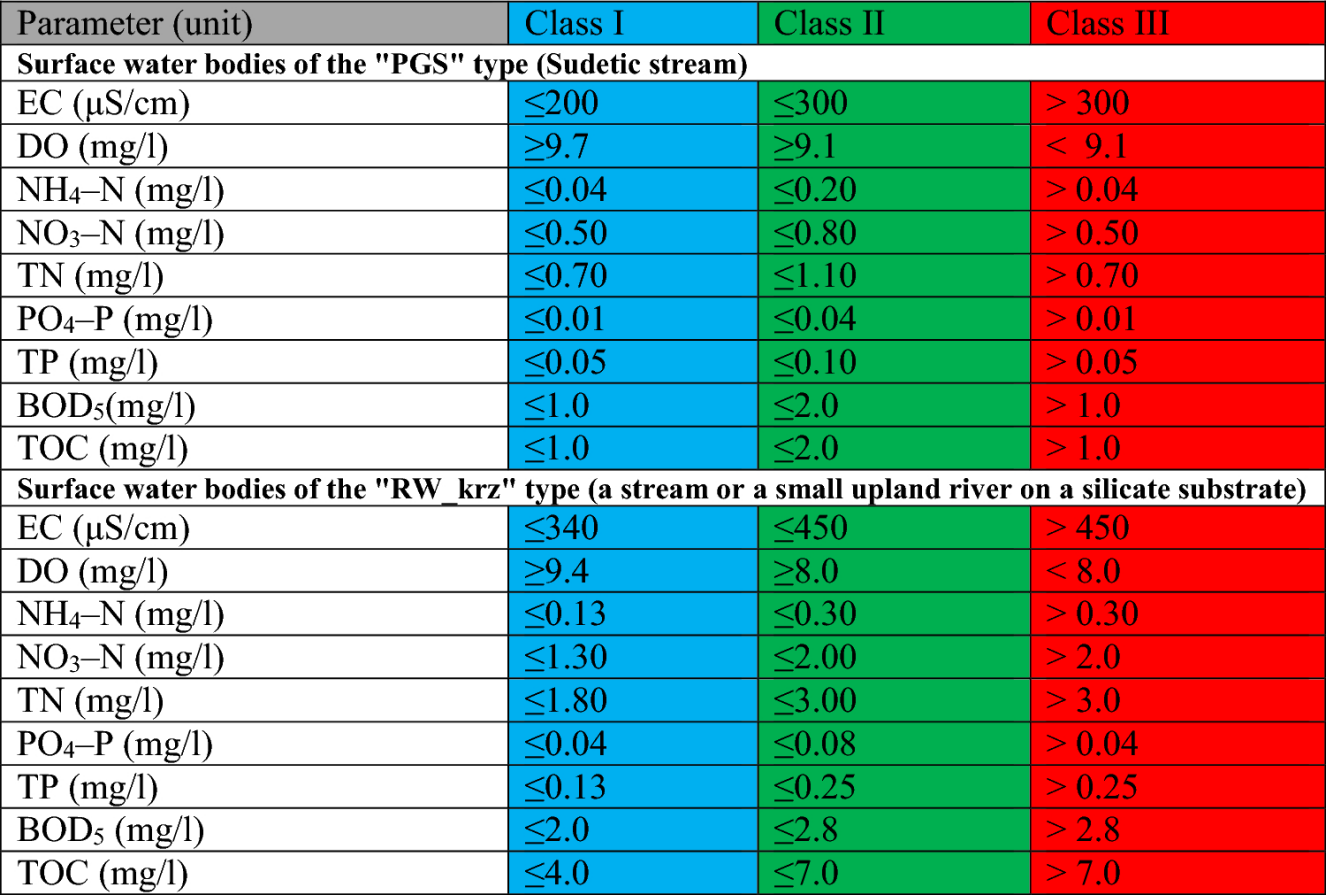
List of limit values of water quality classes for physicochemical parameters of surface water bodies–new classification (points 1–8)^43^.

**Table 2:**
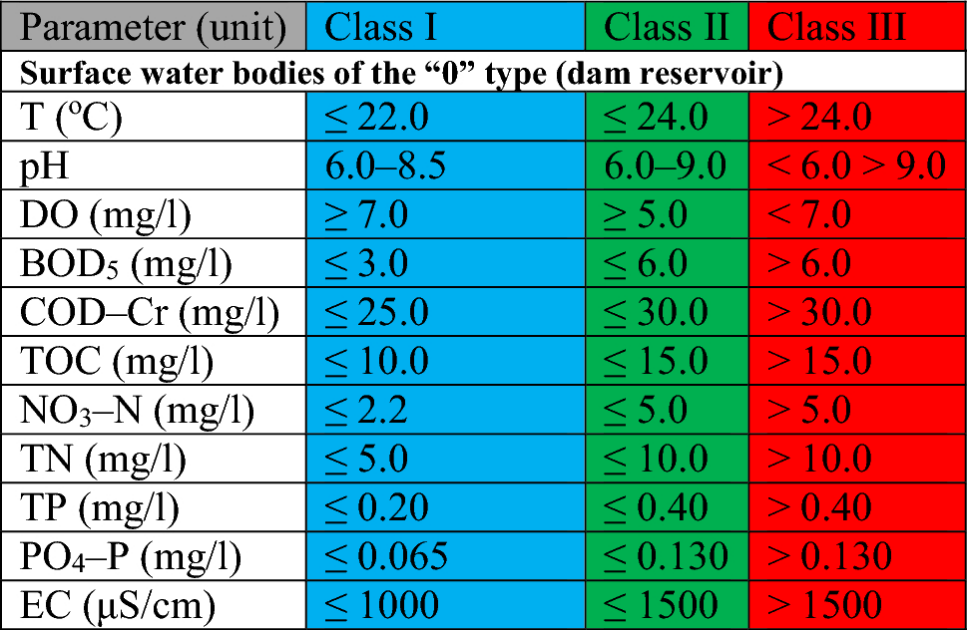
List of limit values of water quality classes for physicochemical parameters of surface water bodies—old classification (points 2, 5, 7, 8)^44^.

The classification of physicochemical elements according to the standardized assessment on a three–point scale was carried out in four steps:

1. Assigning to each tested surface water quality indicator included in the physicochemical elements the appropriate surface water quality class (i.e. class I—very good condition, class II—good condition, class III—condition below good) in relation to the limit values for the types of SWB at the measurement and control points (see Tables 1 and 2);
2. Assigning the number of points to each class, i.e.: I—1 point, II—2 points, III—3 points;
3. Calculating the arithmetic mean based on the assigned values for each parameter;
4. Evaluation of the results according to the scale: 1.00–1.67—very good condition, 1.68–2.33–good condition, 2.34–3.00–condition below good.

In addition, the ecological status was also analyzed in the context of the extent of compliance with the standards imposed by the Water Framework Directive, i.e. achieving a minimum of good status of surface water bodies, which consists of the analyzed ecological status (in this case, physicochemical elements that are an element complementing the assessment), as well as the chemical status focusing on priority substances (important from the point of view of water policy). Therefore, for the purposes of the article, it was assumed that meeting the standard for a given physicochemical element means achieving a minimum of good status (class II) and this standard must be met by at least 80% for the entire analyzed data set, divided into parameters and research points.

For the purposes of the article, statistical analyses were also performed, i.e. basic statistics were determined for parameters at the research points (minimum, maximum, mean, median, standard deviation, coefficient of variation (CV: > 50%—high data variability, 20–50%—medium, < 20%—low)^77^, Lilliefors normality test with histogram analysis (due to the set of over 100 variables for each parameter in the analyzed points; null hypothesis: there is no difference between the observed distribution and the normal distribution). The nonparametric Mann–Whitney U statistical test was also performed, thanks to which the results obtained in reservoirs and rivers were compared, as well as between reservoirs, analyzing them in pairs (the test is based on comparing medians between two sets based on the conducted rank analysis, the data does not have to be normally distributed, all observations in both groups are statistically independent; null hypothesis: symmetry with respect to the probability of a greater value of one of the variables)^22,78^. The values of U and z-ratio were calculated according to the following formulas (1–2):

1 $$U = \frac{{n\left( {n + 1} \right)}}{2}{-} \, \sum_{ranks}$$

2 $$z = \frac{{U - \sigma_{U} }}{{x_{U} }}$$

where n is the number of items in the samples, ∑_ranks_ is the sum of ranks in the sample, σ_U_ is the standard deviation of U, and x_U_ is the mean of U.

For the parameters that most pollute water reservoirs, in the context of failure to meet the desired level of standards, boxplots were also plotted. OriginPro 2024b (OriginLab), SPSS Statistics 26 (IBM) and Statistica 13.3 (StatSoft) were used to perform statistical analyses. The maps were plotted using QGIS 3.34.9 (QGIS Development Team).

### Trophic indices

The analyses also included the calculation of trophic indices, which determine the content of phosphorus and/or nitrogen compounds, which are the main cause of eutrophication in water bodies. They concerned the calculation of two indices: Carlson (TSI_P_) and Trophic Level Index (TLI), which are based on the average content of total phosphorus^45^ (TP, mg/m^3^) and/or total nitrogen (TN, mg/m^3^) for the analyzed research period in all research points. In the case of both parameters, transparency (Secchi disk depth) and chlorophyll^46^ a could also be taken into account, however, due to the small data (N = 22 and 291, respectively), these parameters were omitted, because the results could be unrepresentative. To determine the indices, the following formulas were used:

3 $$TSI_{P} = \, 14.42 \, \log \, \left( {TP} \right) \, + \, 4.15$$

4 $$TLI \, = \, 0.50 \, \left( {TL_{N} + \, TL_{P} } \right)$$

5 $$TL_{N} = \, - 3.61 \, + \, 3.01\log \, \left( {TN} \right)$$

6 $$TL_{P} = \, 0.218 \, + \, 2.92\log \left( {TP} \right)$$

The TSI_P_ classification is presented in Table 3 and the TLI, TLI_N_ and TLI_P_ classification in Table 4.

**Table 3:** Classification of the trophic status of reservoirs based on the Carlson index in terms of phosphorus concentration (TSI_P_)^45,79–81^.

Reference trophic status	Vollenweider (1965)^79^	Sakamoto (1966)^80^	EPA Survey (1974)^81^	Carlson and Simpson (1996)^45^
Oligotrophic	< 10	< 20	< 10	< 6
Oligo–mesotrophic	10–20	–	–	6–12
Mesotrophic	20–50	20–50	10–20	12–24
Mesoeutrophic	50–100	–	–	24–48
Eutrophic	> 100	> 50	> 20	48–96
Hypertrophic	–	–	–	> 96

**Table 4:** Classification of the trophic status using the Trophic Level Index—TLI (the same scale for TLI_N_ and TLI_P_—TLI for total nitrogen and total phosphorus, respectively)^46–48^.

TLI Value	Trophic Status	Description
0.0–1.0	Ultra–microtrophic	Very clean water, with very low levels of nutrients and algae, often glacial water, very good water quality
1.01–2.0	Microtrophic	
2.01–3.0	Oligotrophic	Low levels of nutrients and algae, clear and blue water, good water quality
3.01–4.0	Mesotrophic	Average levels of nutrients and algae, moderate water quality
4.01–5.0	Eutrophic	High amounts of nutrients and algae, cloudy water, poor water quality
5.01–6.0	Supertrophic	Very large amounts of phosphorus and nitrogen, significant algae blooms, poor water transparency, usually not meeting the standards for recreation, very poor water quality
> 6.0	Hypertrophic	

### Water quality indices

The overall water quality was determined based on synthetic water quality indices, which use a number of parameters characterizing, among others, physical, thermal, oxygen and nutrient conditions of water. Six water quality indices were used, namely: Oregon Water Quality Index (OWQI)^58^, Dinius Water Quality Index (DWQI)^59^, Overall Index of Pollution (OIP)^82^, Indian Central Control Board Water Quality Index (CPCB WQI)^83^, Universal Water Quality Index (UWQI)^55^, and The National Sanitation Foundation Water Quality Index (NSF WQI)^51–54^. The indices use raw data, usually based on descriptive statistics (minimum, median, maximum, 10th and 90th percentile). The exception is the NSF WQI, which uses limit values applicable in a given country/region – in this case they are identical to the new water quality classification described in sub sect. “Ecological status”. The aforementioned parameter values are calculated using appropriate formulas, and the final index values are the arithmetic or weighted average of these values, expressed on a scale from 0 to 100. For the purposes of this article, the following point scale was adopted: 0–24.9 points—bad water quality (class V), 25.0–49.9 points—poor water quality (class IV), 50–74.9 points—moderate water quality (class III), 75.0–94.9 points—good water quality (class II), and 95.0–100 points—very good water quality (class I). Details of the index determination are provided in Table S4 in the Supplementary Material^51–55,58,59,82–85^. The method of calculating individual parameters in the indexes depending on their values, as well as the interpretation of results within each of the indexes can be found in the literature.

## Results and discussion

### Basic statistics

This section is described in the Supplementary Material (contains Tables S5 and S6).

### Ecological status

#### Ecological status—points

The average ecological status, examined for 9 indicators according to the new classification of SWB (Table 5), indicates that the parameter responsible for the greatest deterioration of water quality, below good status, in the Dobromierz reservoir (point 2) and Lubachów (point 5) was NO_3_–N (respectively: 2.85 and 2.59). Additionally, in the case of the Dobromierz reservoir, TN (2.82) was responsible for the deterioration of water quality. Therefore, the main pressure for the Dobromierz reservoir is nitrogen compounds of agricultural origin. This is related to the agricultural nature of the catchment area, where the share of agricultural land is over 50%. Nitrogen used to fertilize fields enters the reservoir waters as a result of surface runoff, posing a serious threat to water quality. This is additionally facilitated by the slope of the foothill areas^21^. The situation is different for the Sosnówka reservoir (point 7), where the water condition below good was worsened by oxygen indices, i.e. BOD_5_ (2.73) and TOC (3.0). Increased supplies of organic compounds were most likely responsible for this condition. The standardized average condition in the analyzed points was very good or good and did not differ significantly between the reservoirs (Dobromierz—1.69, Lubachów—1.66, Sosnówka—1.88).

**Table 5:**
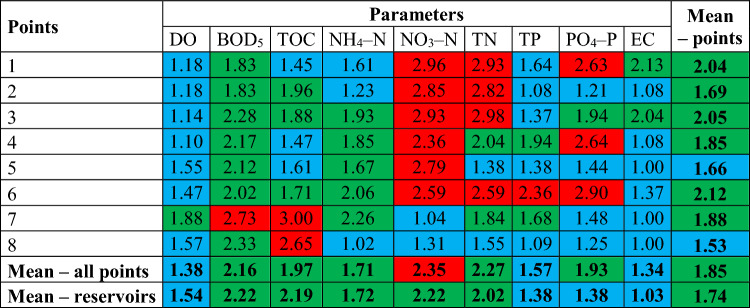
Ecological status of waters according to the new SWB classification for selected control and measurement points according to 9 physicochemical parameters (colors: blue—very good ecological status, green—good, red—below good).

In the remaining control and measurement points, as shown in Table 5, the limit values of the nutrient indicators NO_3_–N, TN, PO_4_–P were also exceeded on the Strzegomka River (points 1 and 3) and on the Bystrzyca River (points 4 and 6). For the Podgórna River, the limit value was exceeded for TOC at the control and measurement point. These indicators are responsible for water eutrophication understood as the enrichment of water with nutrients, in particular nitrogen or phosphorus compounds, causing accelerated growth of algae and higher forms of plant life, as a result of which undesirable disturbances of biological relations in the aquatic environment occur and the quality of these waters deteriorates^62,67,70^. On average, the parameters that most deteriorated the ecological status in all points were NO_3_–N, TN and BOD_5_ (2.35, 2.27 and 2.16). The poorest ecological condition was recorded at points 6, 3 and 1 (2.12, 2.05 and 2.04), but it was within class II.

The average ecological status, examined for 23 indicators according to the old SWB classification, indicates that the deterioration of water quality, below good status, concerns measurement and control points located only on rivers, excluding water reservoirs (Table S8, Supplementary Material). The worst situation occurred on the Strzegomka River, especially at the point above the Dobromierz reservoir (point 1), where exceedances of limit values for nutrient indicators (NO_3_–N, NO_2_–N, TN, PO_4_–P) and salinity indicators (EC, TDS, TA, TH, SO_4_, Cl, Ca, Mg) were recorded. The worst average ecological status was observed in points 1 and 3 (2.16 and 1.75), however, it can still be considered good, in accordance with the requirements of the Water Framework Directive.

Narrowing the analysis of limit values to 9 physicochemical parameters that were assessed in all measurement points according to the old SWB classification, the unfavourable ecological status situation concerns primarily the control and measurement points on rivers located upstream and downstream of reservoirs (Table 6). This concerns the Strzegomka River in terms of biogenic indicators (NO_3_–N = 2.92, TN = 2.87, TN, PO_4_–P = 2.44) and EC (2.77) for point 1 and NO_3_–N (2.66) for point 3. The ecological status below good also concerns points 4 and 6 on the Bystrzyca River in terms of the PO_4_–P parameter with values of 2.36 and 2.74, respectively. Looking at the average for all points, it can be seen that the average ecological status was higher in the reservoirs than in the remaining points (exception: Sosnówka and the point at the outlet on the Podgórna River, however, these are small differences—by 0.03 on the adopted scale).

**Table 6:**
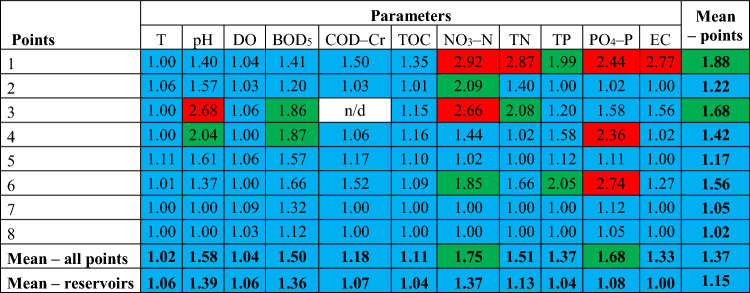
Ecological status of waters according to the old SWB classification for selected control and measurement points according to the currently applicable 11 physicochemical parameters (colors: blue—very good ecological status, green—good, red—below good; n/d—no data).

#### ***Ecological status***—***percentage of standards compliance (minimum good status)***

Analyzing the final average of the averaged values of 9 physicochemical indicators according to the new classification, in most points the indicators do not achieve the 80% compliance with the standard adopted for the purposes of the article (Table 7). Only point 8 on the Podgórna River below the Sosnówka reservoir demonstrates physicochemical parameters that achieve 84.78% of good ecological status of waters. The Sosnówka reservoir demonstrates the lowest percentage of compliance with the standard due to poor values for BOD_5_. The situation is similar at points 1 and 3 on the Strzegomka River (60.48% of the standard and 60.73% of the standard, respectively) due to poor values for NO_3_–N and TN (Table S8, Supplementary Material). In relation to the average for parameters in all points, 5 out of 8 of them do not meet the assumed minimum threshold (NO_3_–N < TN < DO < PO_4_–P < BOD_5_). Looking at the average results for points, in the case of Dobromierz and Lubachów in the water reservoir the ecological status was more often at least good than at the inlet and outlet.

**Table 7:**
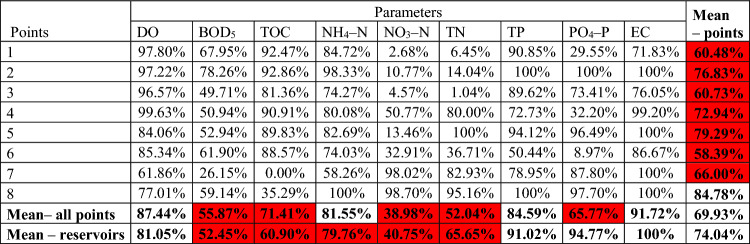
Percentage of compliance with the standard of good ecological status by selected physicochemical indicators at 8 control and measurement points according to the new SWB classification (red: compliance with standards below 80%).

Analyzing the final average of the averaged values of 23 physicochemical indicators according to the old classification, in most points the indicators achieve the 80% compliance with the standard adopted for the purposes of the article (Table S9, Supplementary Material). The exception is point 1 on the Strzegomka River, which shows physicochemical parameters that achieve only 53.34% of the good ecological status of waters (undesirable values, among others, for NO_3_–N, TN, TH, TDS, SO_4_, Cl). A lower percentage of compliance with the standard is also shown by point 3 on the Strzegomka River (73.66%) due to the high percentage of concentration below class II of the ecological status for NO_3_–N and NO_2_–N.

Narrowing down the analysis of average values for 11 physicochemical parameters, common to all points, compliance with 80% of the standard applies to 5 out of 8 analyzed control and measurement points (Table 8). The worst situation occurred at points 1 and 3 on the Strzegomka River, i.e. 64.92% and 72.02% of the standard, respectively, due to frequent exceedances of NO_3_–N, TN and pH values. As can be seen from the results, a higher percentage of compliance with the standards was recorded in water reservoirs than in the remaining points above and/or below them (except for Sosnówka, where the average exceedances were 0.09% higher than in the point below the reservoir).

**Table 8:**
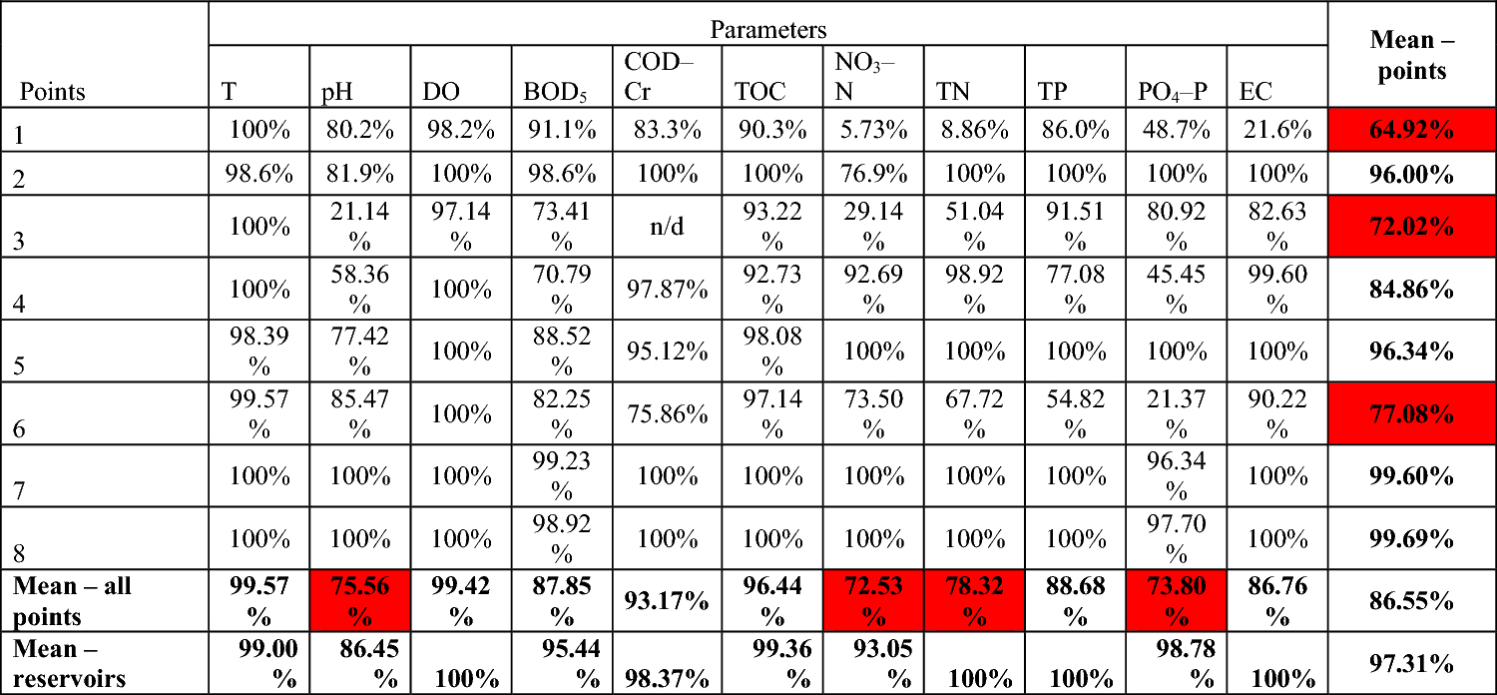
Percentage of compliance with the good ecological status standard by the currently applicable physicochemical indicators at 8 control and measurement points according to the old SWB classification (red: compliance with standards below 80%).

In the context of a wider spatial scale, i.e. Poland, the GIOŚ report from 2020^86^, based on data from 2014 to 2019, showed that 91.6% of river surface water bodies were in poor condition. In relation to physicochemical elements, the most deteriorating parameters were salinity and nutrients (37.7 and 35.6%, respectively). In this case, salinity expressed as EC generally did not show such properties (except for point 1), unlike nutrients (nitrogen and phosphorus compounds), which were largely responsible for the deterioration of the ecological condition at monitoring points. Therefore, remedial actions should be taken to improve water quality.

#### Characteristics of parameters with the lowest percentage of compliance with standards

Referring to the previously described percentages of compliance with the standards, the following parameters were selected for broader comparative analyses concerning, in particular, water reservoirs: NO_3_–N, BOD_5_, NH_4_–N, TOC and TN. They did not reach the threshold value of 80% in at least one of the classifications taken into account for the average of points 2, 5 and 7 (i.e. Dobromierz, Lubachów and Sosnówka reservoirs).

**Fig. 3:**
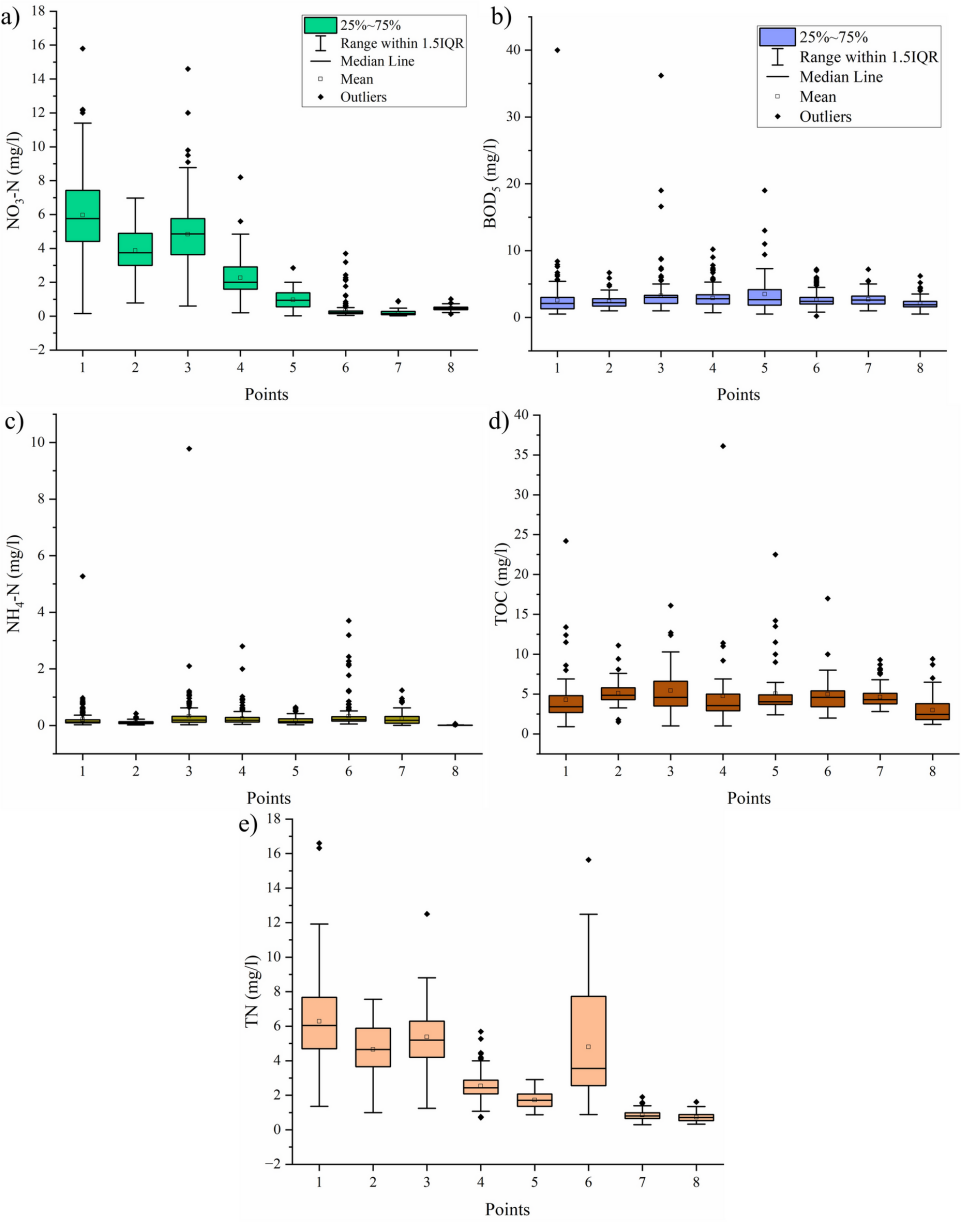
Boxplots for physicochemical parameters at points within the Dobromierz (1–3), Lubachów (4–6) and Sosnówka reservoirs (7–8): (**a**) NO_3_–N, (**b**) BOD_5_, (**c**) NH_4_–N, (**d**) TOC, (**e**) TN.

The lowest percentage of compliance with standards in water reservoirs was recorded for NO_3_–N (66.90%, with 40.75% according to the new classification and 66.90% according to the old one). In relation to water reservoirs themselves, lower compliance with standards is visible for Dobromierz (new classification: 10.77%, old: 76.92%) and Lubachów (new classification: 13.46%, old: 100%) compared to Sosnówka, where the limit values for good status were never exceeded. This situation is illustrated in Fig. 3a, which confirms that NO_3_–N concentrations were clearly higher for Dobromierz, lower in Lubachów, and the lowest—in Sosnówka (medians respectively: 3.75, 0.935 and 0.16 mg/l, the comparison of mean values is analogous). These are statistically significant values, confirmed by the Mann–Whitney U test (p < 0.0001 for each pair of reservoirs). It is worth adding that the highest NO_3_–N concentrations were recorded at points 1 and 3 on the Strzegomka River (medians: above the Dobromierz reservoir – 5.76 mg/l, below the reservoir—4.86 mg/l) and 4 on the Bystrzyca River (median above the Lubachów reservoir—2.00 mg/l). The source of higher NO_3_–N values are runoff from agricultural fields, originating from nitrogen fertilizers and nitrogen discharged together with untreated or insufficiently treated industrial and municipal wastewater. In the catchment area of the Dobromierz and Lubachów reservoirs, a significant share of agricultural land is observed^21,66,67,70^. Intensification of agricultural production brings with it the use of nitrogen fertilizers on a large scale. According to data from the Local Data Bank provided by the Central Statistical Office, the Lower Silesian Voivodeship is among the leaders in the country in terms of nitrogen fertilizer consumption, which is 86.2 kg/ha, compared to the national average of 69.1 kg/ha^87^. The nitrogen load in the waters of Dobromierz and Lubachów is additionally increased by the discharge of domestic and industrial wastewater resulting from the cesspools, so–called septic reservoirs, still located in the catchment area. In the area of the Lubachów reservoir, there are 2,291 cesspools (data for Wałbrzych County), in the area of the Dobromierz reservoir, there are as many as 4,584 cesspools (data for Świdnik County), compared to the average for the province of approx. 3,500 cesspools/county^88^. Due to the lowest degree of canalization in the area of the Lubachów reservoir (only 40%)^63^, there will be situations of direct discharge of wastewater into the soil, especially in rural areas.

The second parameter with the highest exceedances of limit values in reservoirs was BOD_5_ (73.94%; old classification–52.45%, new–95.44%; Fig. 3b). In this case, the situation is reversed than for NO_3_–N, i.e. the lowest average compliance with standards according to the new classification was recorded in Sosnówka, higher—in Lubachów, and the highest—in Dobromierz (respectively: 26.15, 52.94, 78.26%). For the old classification, this order was different, i.e.: Sosnówka > Dobromierz > Lubachów (99.23, 98.55, 88.52%). The second classification may give a better picture of the situation, because all points were classified as this type of SWB (dam reservoirs), and thus the limit values were the same. On the other hand, the streams on which they are located have different characteristics, which speaks in favor of the new classification. In the Lubachów reservoir, the BOD_5_ variability was higher than in Dobromierz and Sosnówka—CV was equal to 86.8, 47.7 and 33.8%, respectively (excluding outliers, i.e. Q_3_–Q_1_, this variability is also visible: 2.3, 1.1 and 1.2 mg/l). The Mann–Whitney U test for pairs of reservoirs proves that in most cases the results are statistically significantly different (exception: Lubachów and Sosnówka). Looking at all points, the highest BOD5 median was observed in point 5, i.e. Lubachów reservoir. This indicates a higher level of organic pollution in this place than in the others, which is manifested by a higher demand for oxygen used by organisms using it. This is confirmed by the presence of recreation centres and summer cottages in the immediate vicinity of the reservoir—a potential source of organic matter^67^.

NH_4_–N is another parameter for which the average compliance with standards for reservoirs was lower than 70% (79.76%, only the new classification; Fig. 3c). The limit values were exceeded most often in the Sosnówka reservoir, followed by Lubachów and Dobromierz (respectively: 41.74, 17.31, 1.67%). This is reflected in the variability of medians, although the limit values for Sosnówka were different than for the other reservoirs due to their classification as different types of SWB (respectively medians: 0.18, 0.135 and 0.096 mg/l). Again, the Mann–Whitney U test showed that statistically significant variability was present between Dobromierz and Lubachów and Dobromierz and Sosnówka. The highest total medians were recorded in points 6, 3, 4 and 7, i.e.: 0.21, 0.19, 0.18 and 0.18 mg/l, which corresponds to the general compliance with standards, everywhere lower than 80%. Higher concentrations of ammonia nitrogen in the context of anthropogenic sources of pollution indicate unregulated water and wastewater management in the catchment area or general human pressure (proximity of urban areas as a source of urea)^52,55,62^. It should be emphasized that the Podgórzyn commune, where the Sosnówka reservoir is located, is the most sewered and has the largest number of cesspools—7171 (data for the Karkonosze district). This is twice as much as the average for the Lower Silesian province^63,88^. Such a large number of non–draining reservoirs constitutes pressure for uncontrolled discharge of wastewater directly into the soil, especially in rural areas, which would translate into an increase in the NH_4_–N content in water. An additional source of NH4–N is the proximity of the urban center of Jelenia Góra (approx. 6 km), with over 75 thousand inhabitants, and the presence of recreation centers and summer cottages in the immediate vicinity of the reservoir^69,70^.

The parameter that did not exceed the standards in more than 80% of cases in reservoirs in the general classification, but in one of them this value was lower, is TOC (new classification: 60.90%, old: 99.36%; Fig. 3d). This case clearly shows that the assignment of the three analyzed research points to different types of SWB (Dobromierz and Lubachów—silicate upland river, Sosnówka—Sudety mountain stream) resulted in the complete failure to meet the standards in Sosnówka in the new classification, while in the other two cases the good condition was noted much more often (Dobromierz: 92.86%, Lubachów: 89.83%). In the old classification, where this type was the same for all reservoirs, Sosnówka managed to meet all the standards, while in Dobromierz and Lubachów—to a lesser extent (respectively: 81.94% and 77.42%). The medians partly reflect this discrepancy, because they do not differ significantly in themselves (Dobromierz—4.85 mg/l, Lubachów—4.04 mg/l, Sosnówka—4.3 mg/l), but the CV coefficient indicates more outliers in Lubachów than in the other two reservoirs (Lubachów—65.6%, Dobromierz—28.6%, Sosnówka—28.2%), which is why the level of compliance with the standards in the uniform classification was the lowest. The highest TOC value was also recorded there (22.5 mg/l). Similarly to BOD_5_, the U–Mann Whitney test did not show statistical significance between the variability of BOD_5_ in the Lubachów and Sosnówka reservoirs. Analysis of medians in all study points shows that the highest values were found in points 2, 3 and 6 (4.85, 4.6 and 4.6 mg/l, respectively), but the overall variability of the collection was not too high in this context (average of medians—3.97 mg/l). As in the case of BOD5, TOC is a measure of the amount of organic compounds in water.

The last parameter taken into account is TN. The situation is similar to TOC, i.e. according to the new classification, the average compliance with standards in reservoirs was not at a satisfactory level, contrary to the old one (respectively: 65.65% and 100.0%; Fig. 3e). There is a similar relationship here as for NO_3_–N, i.e. concentrations were the highest in Dobromierz, intermediate in Lubachów, and the lowest—in Sosnówka (respectively medians: 4.65, 1.705 and 0.805 mg/l). While in the case of the old classification this variability is not visible, because the limit values corresponding to a good state were never exceeded, in the case of the new one—it is. It partly corresponds to the aforementioned median variability, but due to different classification into SWB types in Lubachów this value was higher than in Sosnówka, despite the fact that the concentrations were generally higher (respectively: 100 and 82.93%; on a 3–point scale—1.38 and 1.84, so it was not always a very good condition). In Dobromierz this value was much lower, equal to 14.04%. Each of the reservoirs is different from each other in terms of TN variability—statistical variability was demonstrated in all the analyzed pairs. The points on the Strzegomka River showed a higher median of TN concentrations than in Bystrzyca and Podgórna (the highest in points 1, 3 and 2–6.04, 5.2 and 4.65 mg/l). The exceedances of TN in the Dobromierz reservoir will result from NO_3_–N from agricultural sources causing eutrophication^21,89^, however nitrogen in the form of NO_2_–N cannot be ruled out—in points on the rivers above and below this reservoir the standards were exceeded by 41.67% at the inlet and 83.33% at the outlet.

### Trophic indices

The calculated trophic indices (Table 9) indicate that the general trophic status usually oscillates between mesotrophic and eutrophic. In relation to TSI_P_, point 8 is the most favourable, where 2 out of 4 classifications from Table 4 classify the trophic status as mesotrophic, while according to the remaining two—as eutrophic. The remaining points indicate enrichment with phosphorus compounds in a higher concentration, which qualifies them as mesoeutrophic^79^ or eutrophic^45,80,81^. It is worth noting, however, that the trophic status in this approach is usually more favourable in reservoirs than in rivers (exception: Sosnówka), i.e. 68.04 and 77.27 respectively, however, according to the qualitative classification, this state still does not change. The least enriched in nitrogen are the Sosnówka and Dobromierz reservoirs, while the Lubachów reservoir is more enriched (average multi–year concentrations of TP: 0.069, 0.072 and 0.121 mg/l, respectively).

**Table 9:**
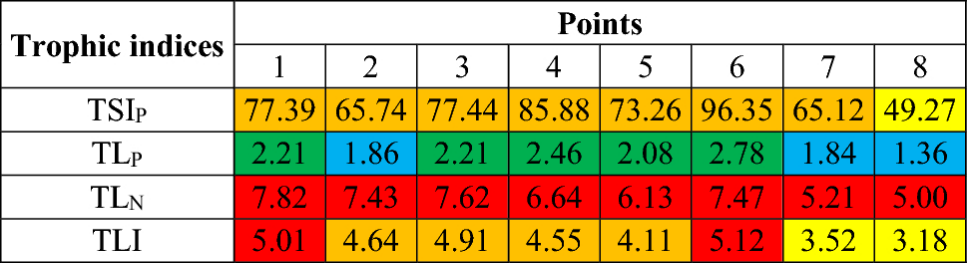
Summary of the results of trophic indices calculated within the Dobromierz (1–3), Lubachów (4–6) and Sosnówka (7–8) reservoirs (colors: (**a**) TSI_P_: < 10—blue, 10–20—green, 20–50—yellow, 50–100—orange, > 100—red; (**b**) TL_P_, TL_N_, TLI: 0.0–2.0—blue, 2.01–3.0—green, 3.01–4.0—yellow, 4.01–5.0—orange, > 5.0—red).

The second analyzed index is TLI. In this case, both the average concentration of TP and TN from the multi–year period were taken into account. The calculations show that the trophic status within the Sosnówka reservoir (points 7 and 8) is mesotrophic, in the Dobromierz and Lubachów reservoirs (points 2 and 5) and below Dobromierz and above Lubachów (points 3 and 4)—eutrophic, while in Strzegomka above Dobromierz and in Bystrzyca below Lubachów—supertrophic (points 1 and 6). The differences in the index values reach even 61% (1.94). When broken down into indexes TL_N_ and TL_P_, it can be seen that nitrogen compounds are more responsible for the deterioration of the trophic status in all cases than phosphorus compounds (average: TL_N_ = 6.67 and TL_P_ = 2.10, respectively), and in qualitative terms, this is a difference of 5 trophic statuss in extreme cases (TL_P_ for points 2 and 7–8—microtrophy; TLN for points 1–6—hypertrophy). Here, too, in water reservoirs, the trophic status is usually better than in rivers (exception: Sosnówka). However, in the case of this analysis, it should be remembered that the average value of the concentration of phosphorus and nitrogen compounds from a multi–year period was taken into account, and these concentrations could change in individual years; the second issue is the results from different years for individual points, so it is also difficult to compare them with each other. The described relationships are therefore more indicative in nature, showing the general trophic status in the analyzed points. In addition, raw water taken from each of the reservoirs is later treated, so its quality delivered to the recipient will be better.

The above analyses of trophic indices confirm that the Dobromierz reservoir is an area sensitive to eutrophication mainly due to nitrogen compounds, especially NO_3_–N, which is also confirmed by the studies of Dąbrowska et al. (2018)^21^, Lejcuś (2004)^90^, Łomotowski et al. (2001)^89^. The reasons for this situation are the intensification of agricultural production in the reservoir catchment area and the lack of effective measures to protect against area pollution. The situation is similar in the case of the Lubachów reservoir, which is also an area sensitive to eutrophication due to nitrogen compounds. This is confirmed by the studies of Kubicz and Cieślar (2016)^66^, who analyzed the water quality of the Bystrzyca River at the inflow and outflow from the reservoir in the period 2000–2006. In their studies, the authors draw attention to the increased values of nitrogen compounds and the high content of TP, the value of which at the outflow affected the deterioration of water quality standards. These are nitrogen and phosphorus compounds originating from agriculture and discharged together with untreated or insufficiently treated industrial and municipal wastewater. Discharges of domestic and economic wastewater may result from the wastewater holding reservoirs still located in the Lubachów catchment area^53^. The third of the analyzed reservoirs, Sosnówka, is an area less sensitive to eutrophication. However, in this case, the pressure is caused by ammonium nitrogen and BOD5. It should be emphasized that the Sosnówka reservoir is the only one of the analyzed reservoirs with the largest share of urbanized areas in the surroundings—a potential source of ammonium nitrogen^70,71^. According to Kubik (2022)^71^, the pressure in the reservoir area is social and economic wastewater from households and economic activities. There is no large, significant industry here and no agricultural wastewater is produced.

Excessive anthropogenic activity, excessive fertilization, industrial and domestic wastewater are the causes of eutrophication^22,91,92^. Eutrophication, especially in fresh waters and water bodies, has become a serious environmental problem, as it is responsible for the degradation of water quality and serious restrictions on its use, especially in the case of water bodies for drinking water purposes^91,93^. Examples of drinking water bodies in the world affected by eutrophication are presented in the Table 10.

**Table 10:** Analysis of the trophic status and water quality indicators of drinking water reservoirs obtained by various researchers.

Reservoir/location	Characteristics of results	Reference
Cedrino/Italy (2010–2011, dane miesięczne)	> 79.6%, probability eutrophic–hypereutrophic conditions and about 20.4% probability oligotrophic–mesotrophic conditions Environmental parameters (TN, NO_2_, NO_3_, NH_4_, TP) and phytoplankton communities indicate a very poor water quality Cause: inflow of nutrients from the catchment in the form of insufficiently treated municipal wastewater and the use of phosphate fertilizers in agricultural areas	Padedda et al., 2017^33^
King Fahd/Saudi Arabia (2010–2012)	Indicators such as nitrogen compounds, TDS, EC, chloride, fluoride and pH showed increasing trends during the October–March period Causes: seasonal rainfall, which generates significant amounts of surface runoff, leading to increased concentrations of pollutants	Chowdhury i Al–Zahrani, 2014^94^
Paldang Reservoir/ South Korea (1996–2019)	The concentrations of TP, TN, BOD, COD, TSS and EC in the reservoir were related to both agricultural activities in the catchment and untreated municipal wastewater. The reservoir indicated a eutrophic status for all seasons	Mamun et al., 2021^95^
Dr. João Penido Reservoir/ Brazil (2011–2013)	Mesotrophic condition, average water quality level in the reservoir Cause: illegal discharge of wastewater from houses near the reservoir	Bucci et al., 2015^96^
Tri An Reservoir/Vietnam (2018–2019)	Slightly eutrophic and hypereutrophic. Water quality was classified as poor in April, June and August and marginal in February, October and December Cause: anthropogenic pollution due to human activity, including nutrients and heavy metals	Pham et al., 2022^97^
Reservoir in Shanghai/ China (2009–2011)	The reservoir is reaching the limit of mesotrophy and eutrophy Increasing values of TN and TP Cause: massive discharges of industrial and municipal wastewater into the river feeding the reservoir	Lu et al., 2015^98^
Nasera/Egypt (2019–2020)	Physicochemical parameters were within the acceptable criteria for drinking water according to the Egyptian drinking water quality standards. A spatial and temporal relationship was observed for the occurrence of higher values of NO_3_, NO_2_, NH_4_, PO_4_, Chl–a and phytoplankton	Goher et al., 2021^28^
Wachusett/USA (2011)	Trophic status: Oligo–mesotrophic reservoir Elevated levels of organic matter in the reservoir, nutrients remain low throughout the year and relatively stable Elevated UV–254 levels lead to increased demand for chlorine and ozone, resulting in the need for higher doses, which increases treatment costs and the formation of disinfection by–products Cause: influx of organic matter after a large rainfall event	Jeznach et al., 2017^99^
Bukówka/Poland (2006–2007)	Increased concentrations of phosphates, BOD_5_, and suspended solids Waters from the Bukówka reservoir area are not sensitive to contamination with nitrogen compounds from agricultural sources. From the point of view of the eutrophication process, the hydrochemical conditions occurring in the Bukówka reservoir catchment area, in terms of the functions performed by the reservoir, are unfavourable for it Assessment of the suitability of water flowing out of the Bukówka reservoir for consumption showed that the limit values of category A3 were exceeded in terms of total suspended solids and BOD_5_ Based on the research, it was found that the Bukówka reservoir catchment area is characterised by low susceptibility to the activation of the load deposited in its area and a small possibility of it reaching the reservoir	Wiatkowski et al., 2015^100^

The authors indicate corrective actions through the creation of models identifying the causes of eutrophication, improving documentation regarding water management in the reservoir and in the catchment, constant monitoring of water quality, limiting the use of nitrogen, using algae–eating zooplankton or removing bottom sediments from the reservoir)^22,33,101,102^. Padedda et al. (2020)^33^ indicate the use of phytoremediation to restore the quality of waters affected by eutrophication. In this case, phytoremediation is understood as the construction of ecological filters for organic components. Phyto–purification systems from algae located directly on the bottom of the river or near it can remove nutrient pollution through uptake by plants.

Lake management strategies should take into account the thermal areas of lakes and morphometric properties, such as maximum depth and height, which significantly affect the concentration of nutrients (nitrogen and phosphorus) responsible for eutrophication^103^. In lakes, nitrogen is present in a more bioavailable form compared to phosphorus^104^. The bioavailability of nitrogen and phosphorus is dependent on biogeochemical processes, i.e. sedimentation, which removes phosphorus and retains it in sediments, and denitrification, which causes nitrogen loss to the atmosphere^105,106^.

Studies by Naderian et al. (2025)^103^ on nutrient limitation in shallow, transitional, and deep lakes showed that regardless of trophic status, phosphorus limitation was dominant in each class, particularly in transitional and deep lakes, followed by nitrogen and phosphorus co-limitation and nitrogen limitation. Phosphorus limitation is dominant in oligotrophic and mesotrophic lakes regardless of lake depth, whereas nitrogen and phosphorus co-limitation is dominant in eutrophic and hypereutrophic lakes regardless of lake depth. The exception is shallow hypereutrophic lakes, where nitrogen is the primary limiting factor. Shallow lakes are more often hypereutrophic than transitional or deep lakes. In contrast, deep lakes are often more oligotrophic than transitional or shallow lakes^103^.

It is important at the stage of designing reservoirs, an effective preventive measure against subsequent eutrophication of the reservoir is the construction of a preliminary reservoir. It retains nutrients and pollutants, and also protects the main reservoir against emergency discharges of wastewater and other substances, thus creating additional space for water storage^107,108^.

As the results show, the Dobromierz, Lubachów and Sosnówka reservoirs belong to the heavily modified water bodies with poor condition, which are at risk of failing to achieve the assumed environmental objectives according to the WFD.

The Odra River Basin Management Plan for 2022–2027^109^ includes measures to reduce trophic pressure in the catchment areas of the analysed reservoirs, including:

- Control activities of the implementation of the nitrate program and related to the use of plant protection products;
- Educational activities for farmers resulting from the “Collection of recommendations for good agricultural practice” or the “Advisory Code of good agricultural practice regarding the reduction of ammonia emissions”,
- Advisory activities aimed at technological advice, helping farmers in applying for financial assistance from EU funds or domestic and foreign funds,
- Activities aimed at reducing the runoff of pollutants from urbanized areas (counteracting erosion or buffer zones).

### Water quality indices

**Fig. 4:**
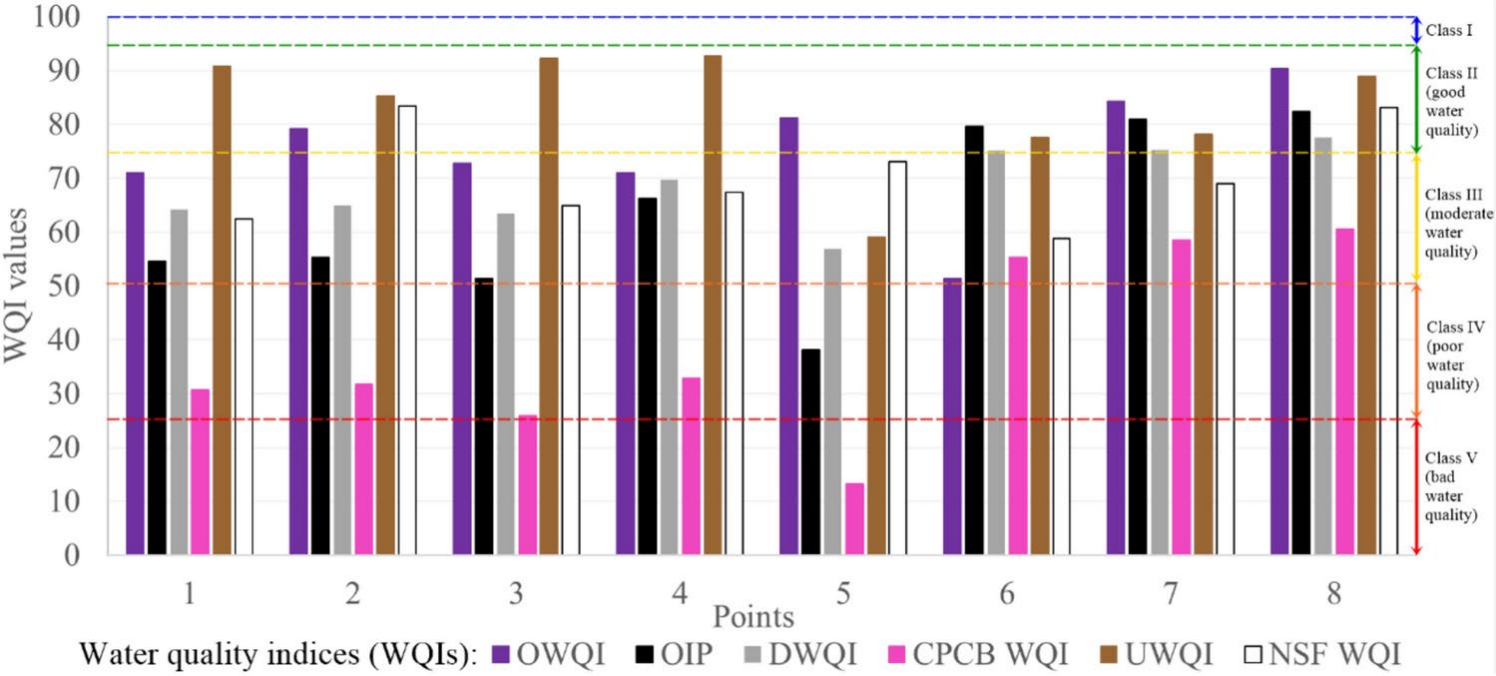
Summary of water quality index results in the assessed measurement and control points within the Dobromierz (1–3), Lubachów (4–6) and Sosnówka (7–8) reservoirs.

As can be seen from Fig. 4, the overall water quality expressed by the average value of the calculated water quality indices is moderate in points 1–6 and good in points 7–8. However, these values differ from each other—the least favourable occurred in the Lubachów reservoir (point 5), and the most favourable—below the Sosnówka reservoir (point 8). Comparing the results from reservoirs and rivers, they are similar to each other, the points on rivers are slightly more favourable (difference of 3.68%; reservoirs—65.29, rivers—67.69).

Looking at the results cross–sectionally, the index showing the weakest results was CPCB WQI (average of points: 38.63, i.e. poor water quality), which takes into account only extreme values of BOD5 and pH, which can significantly distort information about the overall water quality, because over the 30 years of research, there could have been several deviations in values, which, however, should not affect this general picture of the quality status of the water. A solution for drawing more complete conclusions is to use a different approach to the values taken into account in the calculations (e.g. as in OWQI—median value), to take into account a larger number of water quality parameters (as in NSF WQI-6) or to break down the results into individual years (in this case, due to the non–uniformity of conducting measurement series, this solution would be difficult to implement). A justified weighted average, supported by a justification for assigning specific weights to parameters, may also be a more reasonable approach than the arithmetic mean. On the other side of the spectrum is UWQI, which shows that the average overall water quality at the study sites was good (83.14). Here, a wider range of 5 parameters showing oxygen, nutrient and physical conditions was taken into account, and potential errors were minimized by taking into account percentiles of values from the sets. The remaining indices indicate an average moderate water quality at the study sites and their values range from 63.58 (DWQI) to 75.19 (OWQI).

Each of the indices also has its own point scale and corresponding classification, where 5 of the 6 are comprehensive indices, defining the general quality of water, while 1 of them was created to define the suitability of water for different purposes. According to DWQI, these uses are: water supply, recreation, fish, agriculture and industry. Focusing on this index in the context of drinking water, which is the subject of the article, at each of the points it would be necessary to introduce more comprehensive treatment before entering the network (values between 50.1 and 79.9). This means that all water bodies fulfill their function, which is to provide water for consumption. Of the 4 parameters taken into account (BOD5, pH, NO3, water temperature), the most deteriorating were pH, BOD_5_ (respectively 38.75 and 46.02, assuming that each of them would be expressed on a scale of 0 to 100). For other parameters it was respectively: temperature –90.24, NO3—97.36. In the future, it is planned to prepare an article focusing exclusively on water purification technology and assessment of its quality at various stages of this process using the example of the Dobromierz reservoir, so perhaps an attempt will also be made to analyze this index in more detail, taking into account a wider range of analyses, i.e. additionally: dissolved oxygen saturation, the number of Coliform and E. Coli bacteria, alkalinity, hardness, chlorides, specific conductivity and water color.

The authors point out that in the case of surface water intended for consumption, originating from mountain catchments, it is necessary to use different variants of raw water treatment technologies^110^. A large variability of pollutants is observed throughout the year, especially in spring and winter. The quality of raw water from the reservoir is mainly determined by the development of the catchment area. If the catchment area remains under the strong influence of anthropopression and is largely transformed by humans, it should be expected that the retained water will have poor quality values. Incorrectly exploited area around the reservoir will contribute to the outflow of biogenic substances and other pollutants from its area, which will increase the burden of matter on the reservoir and, consequently, the deterioration of water quality and the need to use extensive, expensive water treatment processes^111–113^. This is confirmed by the lowest CPCB WQI value for the Lubachów reservoir (5), which in the catchment structure has a significant share of agricultural and urbanized land, with the least sewered area.

It should be noted that increasingly often in studies, authors also emphasize the polluting influence of rain and climatic conditions on the quality of water in rivers and reservoirs. The ongoing climate change may result in more frequent occurrence of extreme hydrological phenomena, such as floods and droughts^22,114–116^. The analyzed reservoirs Dobromierz, Lubachów and Sosnówka are located in foothill areas, which, during heavy rainfall, poses a risk of rapid surface runoff of water down the river, carrying the load of part of the atmospheric precipitation—an additional source of chlorides in water. The studies by Łomotowski et al. (2001)^89^ conducted on the Dobromierz reservoir indicate that the concentration of chlorides in water, over the 8 years of research, was dependent on the purity of the atmospheric air.

One of the methods of improving the safety of the water supply system is the systematic monitoring of the quality of raw water intended for treatment. It is the quality of water that is one of the main parameters in terms of the fulfilment of the tasks imposed on it by the water supply system. Moreover, properly conducted monitoring of the water taken enables the control and proper conduct of treatment processes, and in the case of incidental situations (high concentrations of pollutants) allows their detection and taking appropriate preventive measures^109,117^.

## Conclusions

The following conclusions can be drawn from the analyses conducted:

1. The use of extended methods of classification of surface water bodies in research points for analyses enables interpretation of changes in the ecological status of waters not only in terms of oxygen and biogenic indicators, but also acidification and salinity. This is extremely important for a full illustration of changes occurring in the catchment area.
2. The assessment of water quality in 3 drinking water reservoirs, made on the basis of water quality indices, for the period 1992–2022, is moderate in the research points for the Dobromierz and Lubachów reservoirs and good for the points within the Sosnówka reservoir. Due to the fact that the analyzed reservoirs are drinking water reservoirs, special attention should be paid to the DWQI index values, which determines the suitability of water for the purposes of drinking water supply. The obtained values of this index, for each of the tested reservoirs, indicate the need to apply comprehensive water treatment before introducing it into the network.
3. The parameters that worsened the ecological status of the reservoirs were: NO_3_-N, TN and PO_4_-P. This indicates strong trophic pressure, the source of which should be sought in the immediate vicinity of the reservoirs. The worst ecological status in relation to the results obtained was achieved at measurement points above and below the Dobromierz reservoir and below the Lubachów reservoir, which is related to the presence of recreational centers and agricultural areas in the immediate vicinity.
4. Nitrogen compounds are more responsible for the trophic status of the analyzed reservoirs than phosphorus compounds (TL_P_ index for points 2, 7 and 8-microtrophy; TL_N_ index for points 1–6-hypertrophy). The general trophic status of the analyzed points usually oscillates between mesotrophic and eutrophic. The analyzed reservoirs are therefore areas at risk of eutrophication.
5. It is recommended to conduct continuous monitoring of quality indicators at permanent measurement points in order to reduce the lack of data, which will allow for the development of adaptation measures to the changes taking place in the catchment area. The current lack of historical data of indicators at research points in individual years disturbs their comparability. Effective assessment of the ecological status of waters should be based on regular measurements of indicator concentrations at permanent measurement points in order to compare them precisely.
6. Future research directions should focus on limiting the surface runoff of nutrients from anthropogenic sources responsible for the eutrophication process of reservoirs. It is the agricultural nature of the reservoir catchment areas and the unorganized water and sewage management in the immediate vicinity of the studied reservoirs that are the main source of pollution with nitrogen and phosphorus compounds.
7. In agricultural catchments, it is additionally recommended to conduct educational activities for farmers in the field of good agricultural practice and the application of the nitrate program.

## Supplementary Information

Supplementary Information.

## Data Availability

All data generated or analyzed during this study are included in this published article.
