# Supplementary material for: Long-term water quality assessment and trophic status trends in Dobromierz, Lubachów and Sosnówka drinking water reservoirs in southwestern Poland
Source: Sci Rep. 2025 Mar 21;15:9804. doi: 10.1038/s41598-025-94219-3 (PMC11928473; doi:10.1038/s41598-025-94219-3)
Supplement: Supplementary file 1 — Supplementary Information. [file 41598_2025_94219_MOESM1_ESM.docx]

**SUPPLEMENTARY MATERIAL**

**Long-Term Water Quality Assessment and Trophic Status Trends in Dobromierz, Lubachów and Sosnówka Drinking Water Reservoirs in southwestern Poland**

Magdalena Szewczyk^1*^, Paweł Tomczyk^2^, Mirosław Wiatkowski^2^

^1^ Provincial Fund for Environmental Protection and Water Management in Opole, Opole, Poland ^2^ Wrocław University of Environmental and Life Sciences, Institute of Environmental Engineering, Wrocław, Poland. *email: m.szewczyk@wfosigw.opole.pl

Table S1. Location of the WIOŚ measurement and control points in Wrocław for the water level in the Dobromierz, Lubachów and Sosnówka reservoirs (SWB types: 4 – Upland silicate stream with coarse–grained substrate – western, RW_krz – stream or small upland river on a silicate substrate, 0 – dam reservoir, L – limnic reservoir, 8 – small silicate upland river – western, PGS – Sudeten stream).

| Name of SWB | Point number in the article | SWB code | SWB type:  old classification / new classification | A year of research |
| --- | --- | --- | --- | --- |
| Strzegomka to the Dobromierz reservoir^1,4^ | 1 | PLRW60004134831 | 4 / RW_krz | 1992–2010, 2012, 2014, 2021 |
| Dobromierz reservoir^1,4^ | 2 | PLRW600001348339 | 0 / L | 2010–2011, 2013–2017, 2020–2021 |
| Strzegomka from the Dobromierz reservoir to Pełcznica^1,4^ | 3 | PLRW60008134859 | 8 / RW_krz | 1992–2008, 2014–2015, 2017, 2021 |
| Bystrzyca to the Lubachów reservoir^2,4^ | 4 | PLRW6000031341959 | 8 / RW_krz | 1992–2008, 2010, 2012, 2014, 2020–2021 |
| Lubachów reservoir^2,4^ | 5 | PLRW6000813439 | 0 / RW_krz | 2010–2011, 2013–2017 |
| Bystrzyca from Piława to Mietków reservoir^2,4^ | 6 | PLRW60004134189 | 8 / PGS | 1992–2006 |
| Sosnówka reservoir^3,4^ | 7 | PLRW600001628889 | 8 / L | 2010–2011, 2013–2015, 2017, 2020–2021 |
| Wrzosówka to Podgórna^3,4^ | 8 | PLRW60003162889 | 0 / PGS | 2000, 2003, 2005–2008, 2010–2015 |

Water samples at designated measurement and control points were collected in plastic or glass bottles and then transported in refrigerated conditions to the laboratory, where they were analyzed within 24 hours of collection^5-7^. Water sample tests were performed based on the methodology according to the standards included in the accreditation of the research laboratory number AB 0758. Sampling equipment consisted of buckets, ladles, watering cans or bottles mounted in a holder of appropriate length. In the case of pH and dissolved oxygen, samples were collected in glass bottles without rinsing them. They were collected up to the cork, without air bubbles. Regarding the determination of parameters, including nitrogen compounds, phosphorus, chlorides, sulphates, BOD_5_, COD, suspensions or conductivity, water was collected in plastic bottles to the brim, with the bottle being rinsed, except for suspensions - water was collected in underfilled bottles. After sampling, the bottle was tightly closed and protected from the effects of light and heating^5-7^. Table S2 presents a summary of laboratory methods for determining physicochemical parameters of water.

Table S2. Specification of laboratory methods for physicochemical determination of water^8^.

| **No.** | **Parameter** | **Name of the Method** | **Measurement Range** |
| --- | --- | --- | --- |
| 1. | pH | Potentiometric method  (PN-EN ISO 10523:2012) | 2.0–12.00 |
| 2. | Electrical conductivity (EC) | Conductometric method  (PN-EN-27888:1999) | 10.0–2000 µS/cm |
| 3. | Temperature of water | Temperature sensor  (PN-C-04584:1977) | 0.0–50.0 °C |
| 4. | Ammonium nitrogen (NH_4_–N) | Continuous low analysis (CFA) method with spectrometric detection (PN-EN ISO 11732:2007) | 0.01–778 mg/L |
| 5. | Nitrate nitrogen (NO_3_–N) | Spectrophotometric method  (PN-82/C-04576.08) | 0.04–225.0 mg/L |
| 6. | Nitrite nitrogen (NO_2_–N) | Spectrophotometric method  (PN-EN 26777:1999) | 0.001–300 mg/L |
| 7. | Total Kjeldahl nitrogen (TKN) | Spectrophotometric method  (PN-EN 25663:2001) | 0.2-100 mg/L |
| 8. | Total nitrogen (TN) | Calculation method  (PB-02/WR from 01.06.2021) | – |
| 9. | Phosphate phosphorus (PO_4_–P) | Spectrometric method  (PN-EN ISO 6878:2006+Ap1:2010+Ap2:2010) | 0.001–0.5 mg/L |
| 10. | Total phosphorus (TP) |  |  |
| 11. | Dissolved oxygen (DO) | Electrochemical sensor  (PN-EN ISO 5814:2013-04) | 0.03–20.00 mg/L |
| 12. | Biochemical oxygen demand (BOD_5_) | Electrochemical method  (PN-EN 1899-2:2002) | 0.1-6.0 mg/L |
| 13. | Chemical oxygen demand (COD) | Spectrophotometric method  (PN-ISO 15705:2005) | 1.0-1000 mg/L |
| 14. | Total suspended solids (TSS) | Weight method  (PN-EN 872:2007+AP1:2007) | 3.0-6000 mg/L |
| 15. | Total dissolved solids (TDS) | Weight method  (PN-EN 15216:2022-03) | 20–10000 mg/L |
| 16. | Total organic carbon (TOC) | Infrared (IR) spectrometry method (PN-EN 1484:1999) | 0.5-1000 mg/L |
| 17. | Total alkalinity (TA) | Titration method  (PN-ISO 6059:1999, PN-EN ISO 9963-1:2001/Ap1:2004) | 5–500 mg/L |
| 18. | Total hardness (TH) | Titration method  (PN-ISO 6059:1999) | 1.1-1000 mg/L |
| 19. | Sulphates (SO_4_) | Ion chromatography method with conductometric detection (IC-CD) (PN-EN ISO 10304-1:2009+AC:2012) | 1–1000 mg/L |
| 20. | Chlorides (Cl) | Ion chromatography method with conductometric detection (IC-CD) (PN-EN ISO 10304-1:2009+AC:2012) | 1–1000 mg/L |
| 21. | Calcium (Ca) | Ion chromatography method with conductometric detection (IC-CD) (PN-EN ISO 14911:2002) | 1–1000 mg/L |
| 22. | Magnesium (Mg) |  | 0.50–1000 mg/L |

Table S3. List of limit values ​​of water quality classes for physicochemical parameters of surface water bodies – old classification (points 1, 3, 4, 6).

| Parameter (unit) | Class I | Class II | Class III |
| --- | --- | --- | --- |
| **SWB type "4" (upland silicate stream with coarse–grained substrate – western)** | | | |
| T (°C) | ≤ 22.0 | ≤ 24.0 | > 24.0 |
| TSS (mg/l) | ≤ 3.0 | ≤ 10.5 | > 10.5 |
| pH | 6.7–8.1 | 6.3–8.1 | < 6.3 > 8.1 |
| DO (mg/l) | ≥ 7.9 | ≥ 7.8 | < 7.8 |
| BOD_5_ (mg/l) | ≤ 2.8 | ≤ 4.5 | > 4.5 |
| COD–Mn (mg/l) | ≤ 6.8 | ≤ 7.5 | > 7.5 |
| COD–Cr (mg/l) | ≤ 12 | ≤ 26 | > 26 |
| TOC (mg/l) | ≤ 4.7 | ≤ 6.2 | > 6.2 |
| NH_4_–N (mg/l) | ≤ 0.03 | ≤ 0.38 | > 0.38 |
| TKN (mg/l) | ≤ 0.4 | ≤ 0.7 | > 0.7 |
| NO_3_–N (mg/l) | ≤ 1.9 | ≤ 2.6 | > 2.6 |
| NO_2_–N (mg/l) | ≤ 0.01 | ≤ 0.03 | > 0.03 |
| TN (mg/l) | ≤ 2.5 | ≤ 3.5 | > 3.5 |
| TP (mg/l) | ≤ 0.08 | ≤ 0.21 | > 0.21 |
| PO_4_–P (mg/l) | ≤ 0.042 | ≤ 0.101 | > 0.101 |
| EC (μS/cm) | ≤ 265 | ≤ 355 | > 355 |
| TDS (mg/l) | ≤ 197 | ≤ 252 | > 252 |
| TA (mg/l) | ≤ 55.0 | ≤ 90.0 | > 90.0 |
| TH (mg/l) | ≤ 128 | ≤ 187 | > 187 |
| SO_4 (mg/l)_ | ≤ 10.9 | ≤ 38.1 | > 38.1 |
| Cl (mg/l) | ≤ 5.0 | ≤ 6.9 | > 6.9 |
| Ca (mg/l) | ≤ 33.9 | ≤ 37.6 | > 37.6 |
| Mg (mg/l) | ≤ 10.8 | ≤ 15.3 | > 15.3 |
| **SWB type "8" (small silicate upland river – western)** | | | |
| T (°C) | ≤ 22.0 | ≤ 24.0 | > 24.0 |
| TSS (mg/l) | ≤ 7.5 | ≤ 13.5 | > 13.5 |
| pH | 7.3–7.7 | 6.6–7.8 | < 6.6 > 7.8 |
| DO (mg/l) | ≥ 7.5 | ≥ 7.4 | < 7.4 |
| BOD_5_ (mg/l) | ≤ 2.4 | ≤ 3.2 | > 3.2 |
| COD–Mn (mg/l) | ≤ 6.9 | ≤ 7.3 | > 7.3 |
| COD–Cr (mg/l) | ≤ 23 | ≤ 27 | > 27 |
| TOC (mg/l) | ≤ 9.1 | ≤ 10.0 | > 10.0 |
| NH_4_–N (mg/l) | ≤ 0.633 | ≤ 0.770 | > 0.770 |
| TKN (mg/l) | ≤ 1.0 | ≤ 1.5 | > 1.5 |
| NO_3_–N (mg/l) | ≤ 2.2 | ≤ 3.7 | > 3.7 |
| NO_2_–N (mg/l) | ≤ 0.01 | ≤ 0.03 | > 0.03 |
| TN (mg/l) | ≤ 4.9 | ≤ 5.2 | > 5.2 |
| TP (mg/l) | ≤ 0.20 | ≤ 0.29 | > 0.29 |
| PO_4_–P (mg/l) | ≤ 0.065 | ≤ 0.101 | > 0.101 |
| EC (μS/cm) | ≤ 404 | ≤ 493 | > 493 |
| TDS (mg/l) | ≤ 282 | ≤ 356 | > 356 |
| TA (mg/l) | ≤ 94.6 | ≤ 119.0 | > 119.0 |
| TH (mg/l) | ≤ 151 | ≤ 206 | > 206 |
| SO_4_ (mg/l) | ≤ 45.0 | ≤ 80.5 | > 80.5 |
| Cl (mg/l) | ≤ 36.2 | ≤ 40.0 | > 40.0 |
| Ca (mg/l) | ≤ 33.9 | ≤ 37.6 | > 37.6 |
| Mg (mg/l) | ≤ 6.9 | ≤ 14.0 | > 14.0 |

Table S4. Basic information on determining the water quality indexes described in the article (OWQI, OIP, DWQI, CPCB WQI, UWQI, and NSF WQI)—equations converted to a scale of index values from 0 to 100 points.

| **Index Name** | **Data for the Index Calculation** | **Parameters Taken into Account** | **Final Index Value** | |
| --- | --- | --- | --- | --- |
|  |  |  | **Equation** | **Explanation of Symbols** |
| Oregon Water Quality Index (OWQI)^9^ | Median value | DO, pH, BOD_5_, NH_4_ + NO_3_, TP, temperature | $OWQI=11.1\sqrt{\frac{n}{\sum_{i=1}^{n} \frac{1}{{SI}_{i}^{2}}}}-111$ | OWQI—the final index value, SI_i_—the sub–index value for each parameter, and n—the number of parameters considered in the calculations. |
| Overall Index of Pollution (OIP)^10^ | Maximum value * | pH, BOD_5_, NO_3_ | $OIP=-6.25 \frac{\sum_{i} P_{i}}{n}+100$ | OIP—the final index value, P_i_—the sub–index value for each parameter, and n—the number of parameters considered in the calculations. |
| Dinius Water Quality Index (DWQI)^11^ | Maximum value * | pH, BOD_5_, temperature, NO_3_ | $DWQI=\sum_{i=1}^{n} I_{i}w_{i}$ | DWQI—the final index value, I_i_—the sub–index value for each parameter, w_i_—the weight value of each parameter (pH = 0.226, BOD_5_ = 0.284, temperature = 0.226, NO_3_ = 0.264), and n—the number of parameters considered in the calculations. |
| Indian Central Pollution Control Board Water Quality Index (CPCB WQI)^12^ | Maximum value * | pH and BOD_5_ | $CPCB WQI=\sum_{i=1}^{n} I_{i}w_{i}$ | CPCB WQI—the final index value. Ii is the sub–index value for each parameter, w_i_—the weight value for each parameter (pH = 0.537, BOD_5_ = 0.463), and n—the number of parameters considered in the calculations. |
| Universal Water Quality Index (UWQI)^13^ | 90th percentile ** | DO, pH, BOD_5_, TP, NO_3_ | $UWQI=\sum_{i=1}^{n} I_{i}w_{i}$ | UWQI—the final index value, I_i—_the sub–index value for each parameter, w_i_—the weight value for each parameter (DO = 0.332, pH = 0.085, BOD_5_ = 0.166, TP = 0.166, NO_3_ = 0.251), and n—the number of parameters considered in the calculations. |
| The National Sanitation Foundation Water Quality Index (NSF WQI)^14-17^ | Raw data | pH, EC,  NO_3_–N,  PO_4_–P, DO, BOD_5_ | *NSF WQI =* $\frac{1}{100}\left[ \sum_{i=1}^{n} q_{i}{\cdot w}_{i} \right]^{2}$ | NSF WQI—the final index value, q_i_—the percentage of samples that fall within the limit values of the parameters, w_i_—the weight value for each parameter (pH = 0.04, EC = 0.13, NO_3_–N = 0.11, PO_4_–P = 0.20, DO = 0.28, BOD5 = 0.24), and n—the number of parameters considered in the calculations. |

Designations in the table: * DO—minimum value; pH—minimum and maximum value (a less favorable result); ** DO—10th percentile; pH—10th and 90th percentile (a less favorable result); NH_4_—ammonia (NH_4_ = 0.78125NH_4_–N); NO_2_—nitrites (NO_2_ = 0.304NO_2_–N); and NO_3_—nitrates (NO_3_ = 0.2257NO_3_–N).

- 1. **Basic statistics**

Analyzing the basic statistics included in Table S5 and Table S6, it can be seen that there was a different variability of the results for individual parameters at the research points and most of them have a non-normal distribution (exception in most points: DO). In most cases, this variability, expressed by the coefficient of variance, was very large (CV > 50%) – for 8 of 12 physicochemical parameters. The average CV at all points was as follows: NH_4_–N > PO_4_–P > TP > COD–Cr > BOD_5_ > T > NO_3_–N > TOC (respectively: 126.7%, 123.45%, 89.50%, 68.9%, 64.1%, 61.5%, 58.1%, 57.1%).

In relation to the water reservoirs themselves, these values ​​are usually smaller, so the parameter values ​​did not change as significantly as in the general set (and thus fewer values ​​are distant from the mean). Nevertheless, in the case of 7 parameters, the average CV was higher than 50%, i.e.: PO_4_–P > NH_4_–N > TP > NO_3_–N > BOD_5_ > T > COD–Cr (respectively: 91.29%, 81.15%, 62.16%, 58.36%, 56.08%, 55.45%, 54.07%). The highest overall variability of results was noted for the Lubachów reservoir, lower for Sosnówka, and the lowest – for Dobromierz (respectively, average CV for all parameters: 52.63%, 52.07%, 38.31%).

Analyzing the median values ​​at points located on rivers and reservoirs, the following relationships are visible: higher water temperature in water reservoirs (points on rivers: 7.7 – 7.85°C, points on reservoirs: 10.8 – 12.2°C), lower oxygen content in water reservoirs (rivers: 10.4 – 11.7 mg/l, reservoirs: 9.65 – 11.0 mg/l). For the remaining parameters, the situation varied depending on the reservoir and the points at the inlet and outlet, so more universal conclusions cannot be drawn on this subject.

In the context of median values ​​for reservoirs, clearly higher EC, COD–Cr, NO_3_–N, TN are visible in Dobromierz than in the other reservoirs (for Dobromierz, Lubachów and Sosnówka, respectively: 311.5, 180 and 95 µS/cm; 13.3, 6.8 and 10.4 mg/l; 3.75, 0.935 and 0.16 mg/l; 4.65, 1.705 and 0.805 mg/l). In the context of median for PO4–P, higher concentrations were recorded in Lubachów and Dobromierz than in Sosnówka (respectively: 0.037, 0.025, 0.001 mg/l). However, in the case of NH_4_–N, the concentrations were the highest in Sosnówka (in Sosnówka, Dobromierz and Lubachów, respectively: 0.18, 0.135 and 0.096 mg/l). For other parameters, the differences in medians were not so clear.

Additionally, the Mann–Whitney U test comparing the results for points located on reservoirs (2, 5, 7) and on rivers (1, 3, 5, 6, 8) proves that at the general level (i.e. comparing the medians in both groups of points and describing them using ranks) most of the values ​​of 12 parameters analyzed within the basic statistics differ statistically significantly for p < 0.05 (exception: BOD_5_). For p < 0.01, this number decreases from 12 to 9 (i.e.: T, DO, TOC,
NH_4_–N, NO_3_–N, TN, TP, PO_4_–P and EC; without pH, BOD_5_ and COD–Cr). The described results are presented in Table S6.

Table S5. Basic statistics for physicochemical parameters used in the assessment of the ecological status of the Dobromierz, Lubachów and Sosnówka reservoirs

| T (°C) | | | | | | | | |
| --- | --- | --- | --- | --- | --- | --- | --- | --- |
|  | N total | Mean | SD | CV | Min | Med. | Max | Q3–Q1 |
| 1 | 224 | 8.17723 | 5.64508 | 0.69034 | 0 | 7.85 | 19.5 | 9.85 |
| 2 | 72 | 11.50556 | 6.78499 | 0.58971 | 0.3 | 10.9 | 24.2 | 12.3 |
| 3 | 172 | 7.56977 | 4.74816 | 0.62725 | 0.1 | 7.8 | 20 | 8.1 |
| 4 | 265 | 8.10189 | 5.5343 | 0.68309 | 0 | 7.8 | 20.2 | 9.8 |
| 5 | 69 | 12.08696 | 6.81257 | 0.56363 | 0.9 | 12.2 | 26 | 11.4 |
| 6 | 230 | 8.34783 | 5.11649 | 0.61291 | 0 | 7.8 | 25 | 9.6 |
| 7 | 118 | 9.91271 | 5.05741 | 0.51019 | 0.5 | 10.8 | 20 | 8.9 |
| 8 | 93 | 8.00968 | 5.12848 | 0.64029 | 0 | 7.7 | 21 | 8.7 |
| pH | | | | | | | | |
| 1 | 227 | 7.98811 | 0.2422 | 0.03032 | 7.2 | 8 | 8.7 | 0.2 |
| 2 | 72 | 8.47222 | 0.51981 | 0.06135 | 7.6 | 8.3 | 10 | 0.9 |
| 3 | 175 | 8.04629 | 0.31836 | 0.03957 | 6.6 | 8 | 9 | 0.3 |
| 4 | 269 | 7.82565 | 0.37123 | 0.04744 | 6.8 | 7.8 | 9.3 | 0.4 |
| 5 | 69 | 8.22319 | 0.91718 | 0.11154 | 7.2 | 7.7 | 10.6 | 1.4 |
| 6 | 234 | 7.56496 | 0.28505 | 0.03768 | 6.7 | 7.6 | 8.5 | 0.3 |
| 7 | 118 | 7.25466 | 0.27561 | 0.03799 | 6.6 | 7.2 | 8.3 | 0.3 |
| 8 | 85 | 7.22824 | 0.42247 | 0.05845 | 5.7 | 7.3 | 8.1 | 0.5 |
| DO (mg/l) | | | | | | | | |
| 1 | 227 | 11.23004 | 1.76026 | 0.15675 | 6.2 | 11.1 | 15.8 | 2.5 |
| 2 | 72 | 11.11486 | 1.71945 | 0.1547 | 6.7 | 11 | 15.1 | 2.25 |
| 3 | 175 | 11.53429 | 1.80159 | 0.15619 | 6.2 | 11.7 | 16.7 | 2.4 |
| 4 | 268 | 11.52724 | 1.59399 | 0.13828 | 7.8 | 11.6 | 16.5 | 2.5 |
| 5 | 69 | 10.60725 | 2.66278 | 0.25103 | 5.3 | 10.7 | 19.4 | 3.4 |
| 6 | 232 | 10.04526 | 2.20611 | 0.21962 | 2.7 | 10.4 | 14 | 2.75 |
| 7 | 118 | 9.80932 | 1.98394 | 0.20225 | 5.7 | 9.65 | 14 | 3.3 |
| 8 | 87 | 10.57356 | 1.94126 | 0.1836 | 6.6 | 10.5 | 14.8 | 2.8 |
| BOD_5_ (mg/l) | | | | | | | | |
| 1 | 237 | 2.55443 | 2.79099 | 1.09261 | 0.5 | 2.1 | 40 | 1.7 |
| 2 | 69 | 2.37681 | 1.13269 | 0.47656 | 1 | 2.2 | 6.7 | 1.1 |
| 3 | 173 | 3.25549 | 3.25043 | 0.99844 | 1 | 3 | 36.2 | 1.2 |
| 4 | 267 | 2.87865 | 1.38885 | 0.48247 | 0.7 | 2.8 | 10.2 | 1.4 |
| 5 | 68 | 3.46985 | 3.01249 | 0.86819 | 0.5 | 2.65 | 19 | 2.3 |
| 6 | 231 | 2.59481 | 1.10393 | 0.42544 | 0.2 | 2.4 | 7.2 | 1 |
| 7 | 130 | 2.69615 | 0.91061 | 0.33774 | 1 | 2.6 | 7.2 | 1.2 |
| 8 | 93 | 2.07097 | 0.92297 | 0.44567 | 0.5 | 1.9 | 6.2 | 0.8 |
| COD–Cr (mg/l) | | | | | | | | |
| 1 | 6 | 24.195 | 32.88864 | 1.35932 | 7.3 | 9.985 | 91 | 7.1 |
| 2 | 37 | 13.53784 | 6.07121 | 0.44846 | 5 | 13.3 | 25.6 | 7.5 |
| 3 | 0 | –– | –– | –– | –– | –– | –– | –– |
| 4 | 47 | 14.53404 | 8.25997 | 0.56832 | 5.9 | 13 | 60 | 7 |
| 5 | 46 | 10.06957 | 8.25208 | 0.81951 | 4 | 6.8 | 47 | 7 |
| 6 | 58 | 21.78966 | 15.80141 | 0.72518 | 6.7 | 16 | 73 | 13.6 |
| 7 | 72 | 10.22472 | 3.6201 | 0.35405 | 2.5 | 10.4 | 20.8 | 3.6 |
| 8 | 40 | 4.948 | 2.69574 | 0.54481 | 2.5 | 4.06 | 12.4 | 4.25 |
| TOC (mg/l) | | | | | | | | |
| 1 | 93 | 4.26538 | 3.05237 | 0.71561 | 0.9 | 3.4 | 24.2 | 2.1 |
| 2 | 70 | 5.09871 | 1.45743 | 0.28584 | 1.5 | 4.85 | 11.1 | 1.5 |
| 3 | 59 | 5.42203 | 2.76991 | 0.51086 | 1 | 4.6 | 16.1 | 3.1 |
| 4 | 55 | 4.75527 | 4.86962 | 1.02405 | 1 | 3.55 | 36.1 | 2.1 |
| 5 | 59 | 5.03898 | 3.30386 | 0.65566 | 2.4 | 4.04 | 22.5 | 1.2 |
| 6 | 35 | 4.97086 | 2.68055 | 0.53925 | 2 | 4.6 | 17 | 2 |
| 7 | 118 | 4.60398 | 1.30223 | 0.28285 | 2.8 | 4.3 | 9.3 | 1.33 |
| 8 | 68 | 2.96824 | 1.64592 | 0.55451 | 1.2 | 2.45 | 9.4 | 2 |
| NH_4_–N (mg/l) | | | | | | | | |
| 1 | 217 | 0.20759 | 0.38392 | 1.84944 | 0.025 | 0.12 | 5.27 | 0.11 |
| 2 | 60 | 0.10756 | 0.07359 | 0.68415 | 0.02 | 0.096 | 0.42 | 0.0665 |
| 3 | 171 | 0.32453 | 0.7733 | 2.38285 | 0.025 | 0.19 | 9.78 | 0.21 |
| 4 | 239 | 0.24431 | 0.25372 | 1.03851 | 0.04 | 0.18 | 2.8 | 0.16 |
| 5 | 52 | 0.18285 | 0.14651 | 0.8013 | 0.025 | 0.135 | 0.64 | 0.139 |
| 6 | 231 | 0.32325 | 0.44177 | 1.36668 | 0.05 | 0.21 | 3.7 | 0.16 |
| 7 | 115 | 0.23711 | 0.225 | 0.94893 | 0.005 | 0.18 | 1.24 | 0.238 |
| 8 | 87 | 0.01032 | 0.01101 | 1.06667 | 0.005 | 0.005 | 0.06 | 0.005 |
| NO_3_–N (mg/l) | | | | | | | | |
| 1 | 227 | 5.96894 | 2.30636 | 0.38639 | 0.16 | 5.76 | 15.8 | 3.02 |
| 2 | 65 | 3.87108 | 1.49535 | 0.38629 | 0.78 | 3.75 | 6.97 | 1.89 |
| 3 | 175 | 4.83663 | 1.89184 | 0.39115 | 0.6 | 4.86 | 14.6 | 2.13 |
| 4 | 260 | 2.26227 | 0.99708 | 0.44075 | 0.2 | 2 | 8.2 | 1.315 |
| 5 | 52 | 0.95804 | 0.55956 | 0.58407 | 0.02 | 0.935 | 2.85 | 0.83 |
| 6 | 231 | 0.32325 | 0.44177 | 1.36668 | 0.05 | 0.21 | 3.7 | 0.16 |
| 7 | 101 | 0.19072 | 0.14882 | 0.78032 | 0.02 | 0.16 | 0.9 | 0.19 |
| 8 | 77 | 0.46925 | 0.14646 | 0.31212 | 0.13 | 0.46 | 1.01 | 0.14 |
| TN (mg/l) | | | | | | | | |
| 1 | 158 | 6.27797 | 2.4132 | 0.38439 | 1.36 | 6.04 | 16.6 | 2.97 |
| 2 | 57 | 4.65175 | 1.56415 | 0.33625 | 1 | 4.65 | 7.56 | 2.23 |
| 3 | 96 | 5.37687 | 1.55338 | 0.2889 | 1.25 | 5.2 | 12.5 | 2.09 |
| 4 | 185 | 2.53978 | 0.78333 | 0.30842 | 0.71 | 2.43 | 5.69 | 0.8 |
| 5 | 34 | 1.72529 | 0.49423 | 0.28646 | 0.87 | 1.705 | 2.91 | 0.71 |
| 6 | 158 | 4.8 | 2.96989 | 0.61873 | 0.88 | 3.55 | 15.64 | 5.17 |
| 7 | 82 | 0.85232 | 0.29567 | 0.3469 | 0.3 | 0.805 | 1.9 | 0.33 |
| 8 | 62 | 0.72758 | 0.24954 | 0.34297 | 0.33 | 0.71 | 1.61 | 0.35 |
| TP (mg/l) | | | | | | | | |
| 1 | 179 | 0.16065 | 0.10658 | 0.66342 | 0.03913 | 0.14 | 0.63 | 0.07 |
| 2 | 39 | 0.07162 | 0.03809 | 0.53186 | 0.02 | 0.06 | 0.2 | 0.03 |
| 3 | 106 | 0.16113 | 0.27245 | 1.69087 | 0.02 | 0.09 | 2.09 | 0.08 |
| 4 | 253 | 0.28938 | 0.34594 | 1.19545 | 0.054 | 0.17 | 3.1 | 0.16 |
| 5 | 34 | 0.12059 | 0.07966 | 0.66056 | 0.02 | 0.09 | 0.39 | 0.09 |
| 6 | 228 | 0.5982 | 0.67322 | 1.12541 | 0.08 | 0.25 | 3.11 | 0.675 |
| 7 | 19 | 0.06858 | 0.04611 | 0.67231 | 0.019 | 0.05 | 0.15 | 0.053 |
| 8 | 46 | 0.02285 | 0.01417 | 0.62024 | 0.0054 | 0.015 | 0.06 | 0.015 |
| PO_4_–P (mg/l) | | | | | | | | |
| 1 | 222 | 0.12405 | 0.10181 | 0.82076 | 0.00669 | 0.10223 | 0.91308 | 0.06196 |
| 2 | 52 | 0.02803 | 0.01505 | 0.53672 | 0.00815 | 0.02511 | 0.07011 | 0.02299 |
| 3 | 173 | 0.09548 | 0.17951 | 1.8801 | 0.01304 | 0.0525 | 1.54898 | 0.04826 |
| 4 | 264 | 0.16684 | 0.15848 | 0.9499 | 0.00978 | 0.11087 | 0.81525 | 0.10321 |
| 5 | 57 | 0.03897 | 0.02162 | 0.5547 | 0.00815 | 0.03652 | 0.12359 | 0.02968 |
| 6 | 234 | 0.44606 | 0.62898 | 1.4101 | 0.03261 | 0.14675 | 2.97729 | 0.19109 |
| 7 | 82 | 0.02032 | 0.03347 | 1.64741 | 0.00326 | 0.00978 | 0.1924 | 0.01467 |
| 8 | 87 | 0.01175 | 0.0244 | 2.07646 | 0.00326 | 0.00652 | 0.17055 | 0.00489 |
| EC (µS/cm) | | | | | | | | |
| 1 | 213 | 409.9155 | 69.85256 | 0.17041 | 176 | 410 | 610 | 95 |
| 2 | 72 | 306.2083 | 32.21055 | 0.10519 | 172 | 311.5 | 374 | 40.5 |
| 3 | 167 | 406.7305 | 84.24015 | 0.20712 | 249 | 382 | 680 | 100 |
| 4 | 251 | 226.0319 | 65.40018 | 0.28934 | 112 | 209 | 520 | 63 |
| 5 | 69 | 180.6957 | 28.71068 | 0.15889 | 123 | 180 | 256 | 41 |
| 6 | 225 | 288.7822 | 166.7247 | 0.57734 | 137 | 228 | 1950 | 131 |
| 7 | 118 | 96.67776 | 12.30986 | 0.12733 | 78 | 95 | 133 | 15.08244 |
| 8 | 93 | 52.06882 | 13.99585 | 0.2688 | 22 | 52 | 129 | 17 |

Table S6. Lilliefors Normality Test for physicochemical parameters used in the assessment of the ecological status of the Dobromierz, Lubachów and Sosnówka reservoirs

| T | | | | |
| --- | --- | --- | --- | --- |
| Points | DF | Statistic | p-value | Decision at level (5%) |
| 1 | 224 | 0.08873 | 2.01E-04 | Reject normality |
| 2 | 72 | 0.10883 | 0.0342 | Reject normality |
| 3 | 172 | 0.11245 | 1.57E-05 | Reject normality |
| 4 | 265 | 0.09391 | 6.60E-06 | Reject normality |
| 5 | 69 | 0.08486 | 0.2 | Can't reject normality |
| 6 | 230 | 0.12616 | 1.70E-09 | Reject normality |
| 7 | 118 | 0.12938 | 4.84E-05 | Reject normality |
| 8 | 93 | 0.12857 | 6.52E-04 | Reject normality |
| pH | | | | |
| 1 | 227 | 0.15978 | 2.00E-15 | Reject normality |
| 2 | 72 | 0.18587 | 1.70E-06 | Reject normality |
| 3 | 175 | 0.11208 | 1.37E-05 | Reject normality |
| 4 | 269 | 0.11119 | 1.49E-08 | Reject normality |
| 5 | 69 | 0.25204 | 7.94E-12 | Reject normality |
| 6 | 234 | 0.12355 | 3.00E-09 | Reject normality |
| 7 | 118 | 0.12111 | 2.14E-04 | Reject normality |
| 8 | 85 | 0.15174 | 4.96E-05 | Reject normality |
| DO | | | | |
| 1 | 227 | 0.05083 | 0.15345 | Can't reject normality |
| 2 | 72 | 0.07289 | 0.2 | Can't reject normality |
| 3 | 175 | 0.05342 | 0.2 | Can't reject normality |
| 4 | 268 | 0.05544 | 0.04507 | Reject normality |
| 5 | 69 | 0.08152 | 0.2 | Can't reject normality |
| 6 | 232 | 0.09405 | 3.58E-05 | Reject normality |
| 7 | 118 | 0.09374 | 0.01266 | Reject normality |
| 8 | 87 | 0.05613 | 0.2 | Can't reject normality |
| BOD_5_ | | | | |
| 1 | 237 | 0.27352 | 8.98E-50 | Reject normality |
| 2 | 69 | 0.13695 | 0.00262 | Reject normality |
| 3 | 173 | 0.30173 | 2.76E-44 | Reject normality |
| 4 | 267 | 0.15058 | 4.25E-16 | Reject normality |
| 5 | 68 | 0.18865 | 2.52E-06 | Reject normality |
| 6 | 231 | 0.149 | 1.49E-13 | Reject normality |
| 7 | 130 | 0.08132 | 0.03464 | Reject normality |
| 8 | 93 | 0.13319 | 3.31E-04 | Reject normality |
| COD-Cr | | | | |
| 1 | 6 | 0.41992 | 0.0014 | Reject normality |
| 2 | 37 | 0.09128 | 0.2 | Can't reject normality |
| 3 | -- | -- | -- | a* |
| 4 | 47 | 0.19115 | 1.73E-04 | Reject normality |
| 5 | 46 | 0.23101 | 1.57E-06 | Reject normality |
| 6 | 58 | 0.20958 | 1.02E-06 | Reject normality |
| 7 | 72 | 0.09332 | 0.12261 | Can't reject normality |
| 8 | 40 | 0.28722 | 5.35E-09 | Reject normality |
| TOC | | | | |
| 1 | 93 | 0.20451 | 2.53E-10 | Reject normality |
| 2 | 70 | 0.11741 | 0.01802 | Reject normality |
| 3 | 59 | 0.1452 | 0.00341 | Reject normality |
| 4 | 55 | 0.26362 | 2.49E-10 | Reject normality |
| 5 | 59 | 0.29412 | 5.56E-14 | Reject normality |
| 6 | 35 | 0.23493 | 3.69E-05 | Reject normality |
| 7 | 118 | 0.16051 | 6.42E-08 | Reject normality |
| 8 | 68 | 0.14365 | 0.00136 | Reject normality |
| NH_4_-N | | | | |
| 1 | 217 | 0.31718 | 6.70E-62 | Reject normality |
| 2 | 60 | 0.16356 | 3.85E-04 | Reject normality |
| 3 | 171 | 0.34925 | 2.48E-59 | Reject normality |
| 4 | 256 | 0.23835 | 2.02E-40 | Reject normality |
| 5 | 52 | 0.18485 | 1.26E-04 | Reject normality |
| 6 | 231 | 0.29837 | 4.24E-58 | Reject normality |
| 7 | 115 | 0.15113 | 8.56E-07 | Reject normality |
| 8 | 87 | 0.37524 | 1.34E-34 | Reject normality |
| NO_3_-N | | | | |
| 1 | 227 | 0.06765 | 0.01352 | Reject normality |
| 2 | 65 | 0.07123 | 0.2 | Can't reject normality |
| 3 | 175 | 0.07074 | 0.03263 | Reject normality |
| 4 | 260 | 0.1679 | 1.06E-19 | Reject normality |
| 5 | 52 | 0.07294 | 0.2 | Can't reject normality |
| 6 | 231 | 0.29837 | 4.24E-58 | Reject normality |
| 7 | 101 | 0.12633 | 4.23E-04 | Reject normality |
| 8 | 77 | 0.11814 | 0.00969 | Reject normality |
| TN | | | | |
| 1 | 158 | 0.07867 | 0.01827 | Reject normality |
| 2 | 57 | 0.08207 | 0.2 | Can't reject normality |
| 3 | 96 | 0.06354 | 0.2 | Can't reject normality |
| 4 | 185 | 0.10856 | 1.55E-05 | Reject normality |
| 5 | 34 | 0.10157 | 0.2 | Can't reject normality |
| 6 | 158 | 0.20858 | 1.31E-18 | Reject normality |
| 7 | 82 | 0.14217 | 3.07E-04 | Reject normality |
| 8 | 62 | 0.09348 | 0.19993 | Can't reject normality |
| TP | | | | |
| 1 | 179 | 0.23039 | 4.38E-26 | Reject normality |
| 2 | 39 | 0.20776 | 1.94E-04 | Reject normality |
| 3 | 106 | 0.34975 | 1.59E-36 | Reject normality |
| 4 | 253 | 0.27297 | 5.56E-53 | Reject normality |
| 5 | 34 | 0.19993 | 0.00138 | Reject normality |
| 6 | 228 | 0.27861 | 1.01E-49 | Reject normality |
| 7 | 19 | 0.19787 | 0.04863 | Reject normality |
| 8 | 46 | 0.27539 | 1.82E-09 | Reject normality |
| PO_4_-P | | | | |
| 1 | 222 | 0.21447 | 5.24E-28 | Reject normality |
| 2 | 52 | 0.15549 | 0.00304 | Reject normality |
| 3 | 173 | 0.32381 | 2.66E-51 | Reject normality |
| 4 | 264 | 0.24743 | 4.27E-45 | Reject normality |
| 5 | 57 | 0.09456 | 0.2 | Can't reject normality |
| 6 | 234 | 0.34884 | 2.22E-81 | Reject normality |
| 7 | 82 | 0.30553 | 3.64E-21 | Reject normality |
| 8 | 87 | 0.36394 | 1.87E-32 | Reject normality |
| EC | | | | |
| 1 | 213 | 0.02697 | 0.2 | Can't reject normality |
| 2 | 72 | 0.09162 | 0.13913 | Can't reject normality |
| 3 | 167 | 0.16089 | 2.14E-11 | Reject normality |
| 4 | 251 | 0.13716 | 2.11E-12 | Reject normality |
| 5 | 69 | 0.06452 | 0.2 | Can't reject normality |
| 6 | 225 | 0.18964 | 6.28E-22 | Reject normality |
| 7 | 118 | 0.09459 | 0.01137 | Reject normality |
| 8 | 93 | 0.09908 | 0.02494 | Reject normality |

Table S7. Results of the Mann–Whitney U test for groups of points on reservoirs (2, 5, 7) and on rivers (1, 3, 5, 6, 8).

| Parameter | Statistic | | |
| --- | --- | --- | --- |
|  | U | Z | p |
| T | 93819.5 | –6.53872 | <0.0001 |
| pH | 141317 | 2.54362 | 0.01097 |
| DO | 153114 | 4.84987 | <0.0001 |
| BOD_5_ | 127968 | –1.06803 | 0.2855 |
| COD–Cr | 13423.5 | 2.22461 | 0.02611 |
| TOC | 26924 | –6.02139 | <0.0001 |
| NH_4_–N | 122431 | 2.84732 | 0.00441 |
| NO_3_–N | 146572 | 8.92264 | <0.0001 |
| TN | 84261.5 | 9.68926 | <0.0001 |
| TP | 59181.5 | 9.20144 | <0.0001 |
| PO_4_–P | 161622.5 | 15.91309 | <0.0001 |
| EC | 185157 | 12.51162 | <0.0001 |

| Points | Parameters | | | | | | | | | | | | | | | | | | | | | | | **Mean  – points** |
| --- | --- | --- | --- | --- | --- | --- | --- | --- | --- | --- | --- | --- | --- | --- | --- | --- | --- | --- | --- | --- | --- | --- | --- | --- |
|  | T | TSS | pH | DO | BOD_5_ | COD–Mn | COD–Cr | TOC | NH_4_–N | TKN | NO_3_–N | NO_2_–N | TN | TP | PO_4_–P | EC | TDS | TA | TH | SO4 | Cl | Ca | Mg |  |
| 1 | 1.00 | 1.84 | 1.40 | 1.04 | 1.41 | 1.12 | 1.50 | 1.35 | 2.09 | 2.04 | 2.92 | 2.34 | 2.87 | 1.99 | 2.44 | 2.77 | 2.83 | 2.79 | 2.82 | 2.95 | 2.99 | 2.78 | 2.48 | **2.16** |
| 2 | 1.06 | n/a | 1.57 | 1.03 | 1.20 | n/a | 1.03 | 1.01 | n/a | n/a | 2.09 | n/a | 1.40 | 1.00 | 1.02 | 1.00 | n/a | n/a | n/a | n/a | n/a | n/a | n/a | **1.22** |
| 3 | 1.00 | 1.68 | 2.68 | 1.06 | 1.86 | 1.07 | n/d | 1.15 | 1.15 | 1.20 | 2.66 | 2.81 | 2.08 | 1.20 | 1.58 | 1.56 | 1.89 | 2.28 | 2.51 | 1.98 | 1.06 | 1.80 | 2.20 | **1.75** |
| 4 | 1.00 | 1.60 | 2.04 | 1.00 | 1.87 | 1.09 | 1.06 | 1.16 | 1.06 | 1.32 | 1.44 | 2.08 | 1.02 | 1.58 | 2.36 | 1.02 | 1.04 | 1.16 | 1.08 | 1.43 | 1.05 | 1.03 | 1.41 | **1.34** |
| 5 | 1.11 | n/a | 1.61 | 1.06 | 1.57 | n/a | 1.17 | 1.10 | n/a | n/a | 1.02 | n/a | 1.00 | 1.12 | 1.11 | 1.00 | n/a | n/a | n/a | n/a | n/a | n/a | n/a | **1.17** |
| 6 | 1.01 | 1.61 | 1.37 | 1.00 | 1.66 | 1.09 | 1.52 | 1.09 | 1.13 | 1.49 | 1.85 | 2.24 | 1.66 | 2.05 | 2.74 | 1.27 | 1.28 | 1.10 | 1.06 | 1.57 | 1.36 | 1.03 | 1.44 | **1.46** |
| 7 | 1.00 | n/a | 1.00 | 1.09 | 1.32 | n/a | 1.00 | 1.00 | n/a | n/a | 1.00 | n/a | 1.00 | 1.00 | 1.12 | 1.00 | n/a | n/a | n/a | n/a | n/a | n/a | n/a | **1.05** |
| 8 | 1.00 | n/a | 1.00 | 1.03 | 1.12 | n/a | 1.00 | 1.00 | n/a | n/a | 1.00 | n/a | 1.00 | 1.00 | 1.05 | 1.00 | n/a | n/a | n/a | n/a | n/a | n/a | n/a | **1.02** |
| **Mean**  **– all points** | **1.02** | **1.68** | **1.58** | **1.04** | **1.50** | **1.09** | **1.18** | **1.11** | **1.36** | **1.51** | **1.75** | **2.37** | **1.51** | **1.37** | **1.68** | **1.33** | **1.76** | **1.83** | **1.87** | **1.98** | **1.62** | **1.66** | **1.88** | **1.55** |
| **Mean**  **– reservoirs** | **1.06** | **n/a** | **1.39** | **1.06** | **1.36** | **n/a** | **1.07** | **1.04** | **n/a** | **n/a** | **1.37** | **n/a** | **1.13** | **1.04** | **1.08** | **1.00** | **n/a** | **n/a** | **n/a** | **n/a** | **n/a** | **n/a** | **n/a** | **1.15** |

Table S8. Ecological status of waters according to the old JCWP classification for selected control and measurement points for 23 physicochemical parameters.

Table S9. Percentage of compliance with the good ecological status standard by selected physicochemical indicators at 8 control and measurement points according to the old JCWP classification.

| Points | Parameters | | | | | | | | | | | | | | | | | | | | | | | **Mean**  **– points** |
| --- | --- | --- | --- | --- | --- | --- | --- | --- | --- | --- | --- | --- | --- | --- | --- | --- | --- | --- | --- | --- | --- | --- | --- | --- |
|  | T | TSS | pH | DO | BOD_5_ | COD–Mn | COD–Cr | TOC | NH_4_–N | TKN | NO3–N | NO2–N | TN | TP | PO_4_–P | EC | TDS | TA | TH | SO_4_ | Cl | Ca | Mg |  |
| 1 | 100.00% | 82.23% | 80.18% | 98.24% | 91.14% | 94.65% | 83.33% | 90.32% | 89.86% | 72.61% | 5.73% | 58.33% | 8.86% | 86.03% | 48.65% | 21.60% | 14.98% | 19.83% | 16.83% | 4.71% | 0.56% | 15.63% | 42.42% | **53.34%** |
| 2 | 98.61% | n/a | 81.94% | 100.00% | 98.55% | n/a | 100.00% | 100.00% | n/a | n/a | 76.92% | n/a | 100.00% | 100.00% | 100.00% | 100.00% | n/a | n/a | n/a | n/a | n/a | n/a | n/a | **96.00%** |
| 3 | 100.00% | 89.33% | 21.14% | 97.14% | 73.41% | 96.50% | n/d | 93.22% | 93.57% | 94.79% | 29.14% | 16.67% | 51.04% | 91.51% | 80.92% | 82.63% | 79.41% | 57.02% | 45.54% | 85.71% | 97.86% | 64.00% | 80.00% | **73.66%** |
| 4 | 100.00% | 88.00% | 58.36% | 100.00% | 70.79% | 96.02% | 97.87% | 92.73% | 97.27% | 92.59% | 92.69% | 79.27% | 98.92% | 77.08% | 45.45% | 99.60% | 98.80% | 95.07% | 100.00% | 99.58% | 98.39% | 98.53% | 98.53% | **90.24%** |
| 5 | 98.39% | n/a | 77.42% | 100.00% | 88.52% | n/a | 95.12% | 98.08% | n/a | n/a | 102.22% | n/a | 100.00% | 100.00% | 100.00% | 100.00% | n/a | n/a | n/a | n/a | n/a | n/a | n/a | **96.34%** |
| 6 | 99.57% | 85.78% | 85.47% | 100.00% | 82.25% | 95.73% | 75.86% | 97.14% | 94.81% | 85.31% | 73.50% | 65.52% | 67.72% | 54.82% | 21.37% | 90.22% | 91.27% | 99.05% | 99.21% | 98.28% | 82.68% | 98.59% | 99.53% | **84.51%** |
| 7 | 100.00% | n/a | 100.00% | 100.00% | 99.23% | n/a | 100.00% | 100.00% | n/a | n/a | 100.00% | n/a | 100.00% | 100.00% | 96.34% | 100.00% | n/a | n/a | n/a | n/a | n/a | n/a | n/a | **99.60%** |
| 8 | 100.00% | n/a | 100.00% | 100.00% | 98.92% | n/a | 100.00% | 100.00% | n/a | n/a | 100.00% | n/a | 100.00% | 100.00% | 97.70% | 100.00% | n/a | n/a | n/a | n/a | n/a | n/a | n/a | **99.69%** |
| **Mean – all points** | **99.57%** | **86.34%** | **75.56%** | **99.42%** | **87.85%** | **95.72%** | **93.17%** | **96.44%** | **93.87%** | **86.33%** | **72.53%** | **54.95%** | **78.32%** | **88.68%** | **73.80%** | **86.76%** | **71.11%** | **67.74%** | **65.40%** | **72.07%** | **69.87%** | **69.19%** | **80.12%** | **81.08%** |
| **Mean – reservoirs** | **99.00%** | **n/a** | **86.45%** | **100.00%** | **95.44%** | **n/a** | **98.37%** | **99.36%** | **n/a** | **n/a** | **93.05%** | **n/a** | **100.00%** | **100.00%** | **98.78%** | **100.00%** | **n/a** | **n/a** | **n/a** | **n/a** | **n/a** | **n/a** | **n/a** | **97.31%** |

References

1. Wody Polskie. Karta charakterystyki JCWP zbiornik Dobromierz (2022).
2. Wody Polskie. Karta charakterystyki JCWP Bystrzyca do zbiornika Lubachów (2022).
3. Wody Polskie. Karta charakterystyki JCWP Bystrzyca do zbiornika Sosnówka (2022).
4. WIOŚ Wrocław – Wojewódzki Inspektorat Ochrony Środowiska we Wrocławiu. Ocena stanu jednolitych części wód powierzchniowych na terenie województwa dolnośląskiego za rok 2017. Wrocław (2018).
5. Wdowczyk, A., Szymańska–Pulikowska, A., Domańska, M. Analysis of the Bacterial Biocenosis of Activated Sludge Treated with Leachate from Municipal Landfills. Int. J. Environ. Res. Public Health. 19(3), 1801. https://doi.org/10.3390/ijerph19031801 (2022).
6. Wdowczyk, A., Szymańska–Pulikowska, A. Micro– and Macroelements Content of Plants Used for Landfill Leachate Treatment Based on Phragmites australis and Ceratophyllum demersum. Int. J. Environ. Res. Public Health. 19(10), 6035. https://doi.org/10.3390/ijerph19106035 (2022).
7. Szymańska–Pulikowska, A., Wdowczyk, A. Changes of a Landfill Leachate Toxicity as a Result of Treatment With Phragmites australis and Ceratophyllum demersum – A Case Study. Front. Environ. Sci. 9, 739562. doi: 10.3389/fenvs.2021.739562 (2021).
8. Polskie Centrum Akredytacji. Zakres Akredytacji Laboratorium Badawczego nr AB 075 (2021).
9. Cude, C. G. Oregon Water Quality Index: A tool for evaluating water quality management efectiveness’ quality issues by the public and policy makers. JAWRA J. Am. Water Resour. Assoc. 37(1), 125–137. https://doi.org/10.1111/j.1752–1688.2001.tb05480.x (2001).
10. Dinius, S.H. Design of an Index of Water Quality, JAWRA. 23(5), 833–843. https://doi.org/10.1111/j.1752–1688.1987.tb02959 (1987).
11. Vilanova, M.R.N., Filho, P.M., Perrella Balestieri, J.Z. Performance measurement and indicators for water supply management: Review and international cases. Renewable and sustainable energy reviews 43, 1–12, https://doi.org/10.1016/j.rser.2014.11.043 (2015).
12. Sargaonkar, A., Deshpande, V. Development of an overall index of pollution for surface water based on a general classification scheme in Indian context, Environ. Monit. Assess. 89(1), 43–67. https://doi.org/10.1023/A:1025886025137 (2003).
13. Boyacioglu, H. Development of a water quality index based on a European classification scheme. Water SA 33(1), 101–106. https:// doi. org/ 10. 4314/ wsa. v33i1. 47882 (2007).
14. House, M. A. A water quality index for river management. Water Environ. J. 3(4), 336–344. https:// doi. org/ 10. 1111/J. 1747– 6593.1989. TB015 38.X (1989).
15. Bordalo, A. A., Nilsumranchit, W. & Chalermwat, K. Water quality and uses of the Bangpakong river (Eastern Thailand). Water Res. 35(15), 3635–3642. https:// doi. org/ 10. 1016/ S0043– 1354(01) 00079–3 (2001).
16. Breabăn, I. G., Gheţeu, D. & Mădălina, P. A. I. U. Determination of water quality index of Jijia and Miletin ponds. Bull. University of agricultural sciences and veterinary medicine Cluj–Napoca. Agriculture 69(2), 160–167 (2012).
17. Cymes, I.. Glińska–Lewczuk, K. The use of water quality indices (WQI and SAR) for multipurpose assessment of water in dam reservoirs. J. Elementol. 21(4), 1211–1224. https:// doi. org/ 10. 5601/ JELEM. 2016. 21.2. 1200 (2016).
